# Phylogenetic analysis of cell-cycle regulatory proteins within the Symbiodiniaceae

**DOI:** 10.1038/s41598-020-76621-1

**Published:** 2020-11-24

**Authors:** Lucy M. Gorman, Shaun P. Wilkinson, Sheila A. Kitchen, Clinton A. Oakley, Arthur R. Grossman, Virginia M. Weis, Simon K. Davy

**Affiliations:** 1 School of Biological Sciences, Victoria University of Wellington, Wellington, 6140 New Zealand; 2 Division of Biology and Biological Engineering, California Institute of Technology, Pasadena, CA 91125 USA; 3 Department of Plant Biology, The Carnegie Institution for Science, Stanford, CA 94305 USA; 4 Department of Integrative Biology, Oregon State University, Corvallis, OR 97331 USA

**Keywords:** Evolutionary biology, Marine biology

## Abstract

In oligotrophic waters, cnidarian hosts rely on symbiosis with their photosynthetic dinoflagellate partners (family Symbiodiniaceae) to obtain the nutrients they need to grow, reproduce and survive. For this symbiosis to persist, the host must regulate the growth and proliferation of its symbionts. One of the proposed regulatory mechanisms is arrest of the symbiont cell cycle in the G_1_ phase, though the cellular mechanisms involved remain unknown. Cell-cycle progression in eukaryotes is controlled by the conserved family of cyclin-dependent kinases (CDKs) and their partner cyclins. We identified CDKs and cyclins in different Symbiodiniaceae species and examined their relationship to homologs in other eukaryotes. Cyclin proteins related to eumetazoan cell-cycle-related cyclins A, B, D, G/I and Y, and transcriptional cyclin L, were identified in the Symbiodiniaceae, alongside several alveolate-specific cyclin A/B proteins, and proteins related to protist P/U-type cyclins and apicomplexan cyclins. The largest expansion of Symbiodiniaceae cyclins was in the P/U-type cyclin groups. Proteins related to eumetazoan cell-cycle-related CDKs (CDK1) were identified as well as transcription-related CDKs. The largest expansion of CDK groups was, however, in alveolate-specific groups which comprised 11 distinct CDK groups (CDKA-J) with CDKB being the most widely distributed CDK protein. As a result of its phylogenetic position, conservation across Symbiodiniaceae species, and the presence of the canonical CDK motif, CDKB emerged as a likely candidate for a *Saccharomyces cerevisiae* Cdc28/Pho85-like homolog in Symbiodiniaceae. Similar to cyclins, two CDK-groups found in Symbiodiniaceae species were solely associated with apicomplexan taxa. A comparison of *Breviolum minutum* CDK and cyclin gene expression between free-living and symbiotic states showed that several alveolate-specific CDKs and two P/U-type cyclins exhibited altered expression *in hospite*, suggesting that symbiosis influences the cell cycle of symbionts on a molecular level. These results highlight the divergence of Symbiodiniaceae cell-cycle proteins across species. These results have important implications for host control of the symbiont cell cycle in novel cnidarian–dinoflagellate symbioses.

## Introduction

Many cnidarians in the marine environment, including reef-building corals, form symbiotic relationships with photosynthetic dinoflagellates from the family Symbiodiniaceae^1^. These dinoflagellate symbionts are located in host gastrodermal cells inside symbiosomes (vacuoles consisting of a host-derived membrane)^2^. This closely integrated intracellular relationship indicates that symbiont population maintenance by the host was likely integral to the evolution of the symbiosis^1,3^. To date, most studies examining symbiont cell division *in hospite* have focused on nutrient availability^4–9^. However, symbiont growth rate appears to be controlled by more than nutrient limitation, as nutrient-replete symbionts *in hospite* still have a growth rate that is less than 20% of symbionts *ex hospite*^5^.

Besides nutrient control, other proposed host regulatory mechanisms of symbiont growth and proliferation include pre-mitotic cell-cycle control and post-mitotic autophagy, expulsion and apoptosis^1,7,10,11^. However, the contribution of each mechanism towards the regulation of symbiont biomass, from the onset to the stabilisation of the symbiosis, is unknown. Smith and Muscatine^7^ proposed that the main control of a dampened symbiont growth rate *in hospite* is from the cnidarian host arresting the cell cycle of its resident symbionts. In the eukaryotic cell cycle there is one quiescent stage (G_0_) and four subsequent cycling phases: G_1_ (gap 1) where cells grow and are sensitive to extracellular cues such as growth factors^12^; S (synthesis) where genomic DNA is replicated and synthesised^13^; G_2_ (gap 2), where DNA breaks that occur during the S phase are repaired before mitosis^14^; and M (mitosis), where two equal copies of the chromosomes are distributed between the two cells^13^. In the sea anemone *Exaiptasia pallida* (‘Aiptasia’), 80% of the resident symbionts were shown to be arrested at the G_0_/G_1_ phase compared with 40–55% in culture^7^.

**Figure 1:**
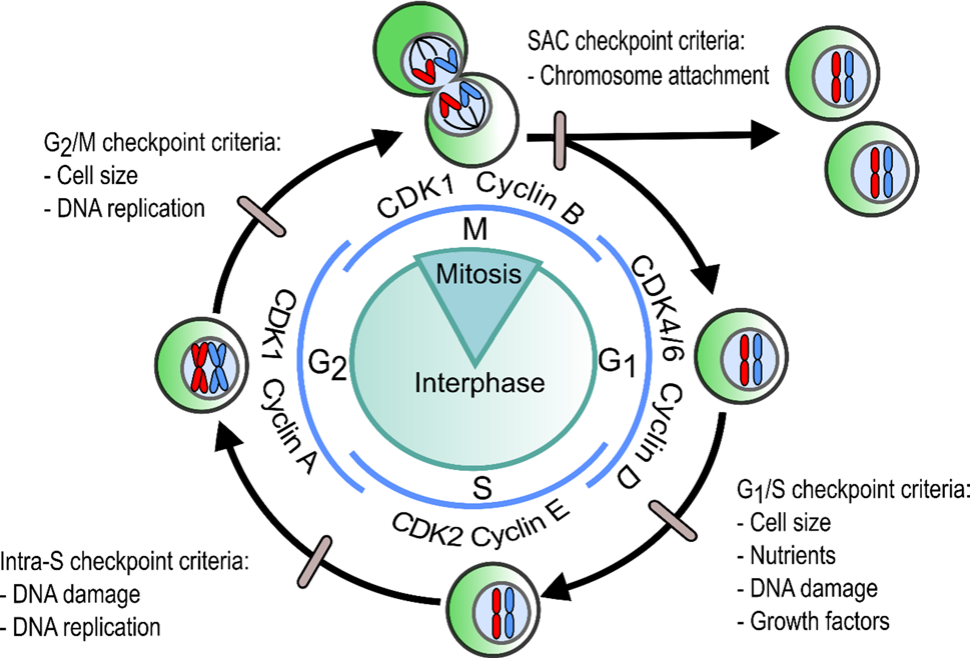
The generalised cell cycle in eumetazoans illustrating the cell-cycle phase and regulatory checkpoints (grey ovals) with their complementary criteria that must be met for the cell cycle to progress through that checkpoint. Blue lines depict the formation of cell-cycle phase associated CDK-cyclin complexes in *Homo sapiens*. *SAC* Spindle Assembly Checkpoint.

Once a cell enters the cell cycle, it can be arrested at a series of cell-cycle checkpoints (Fig. 1). These checkpoints monitor the integrity and correct progression of the cell cycle with each checkpoint containing criteria that must be met for a cell to progress to the next stage of the cycle^15,16^. Each checkpoint is regulated by cyclin-dependent kinases (CDKs) and their partner cyclins^17^. Once a cell meets its checkpoint criteria, cyclins are synthesised and bind to their partner CDKs^17^. Cyclins regulate the catalytic activity of CDKs^18^. These CDK-cyclin complexes can directly trigger cell-cycle progression (Fig. 1) or indirectly trigger cell-cycle progression through a variety of other downstream events such as transcription, DNA damage repair, proteolytic degradation and metabolism^19^. Table S1 summarises the cell-cycle stage and roles of individual CDK and cyclin proteins. CDK-cyclin complexes in *Homo sapiens* are shown in Fig. 1; however, the type and quantity of CDKs and cyclins are specific to a particular species^17^.

Identification of cell-cycle proteins in the Symbiodiniaceae is just beginning, with a study by Cato et al.^20^ finding 10 distinct CDKs and 15 distinct cyclin genes in the genome of *Breviolum minutum*. In the same study^20^, qPCR analysis revealed that a cyclin B2/CDK1 pair was expressed during the G_1_/S phase transition in cultured *B. minutum*. As there are at least nine genera of Symbiodiniaceae^21,22^, determining whether cell-cycle proteins present in *B. minutum* are conserved across the Symbiodiniaceae will inform our understanding of cell-cycle progression and cellular growth rates in this family. For example, a recent study^23^ comparing cell-cycle progression between four Symbiodiniaceae genera (*Symbiodinium*, *Breviolum*, *Cladocopium* and *Durusdinium*) in culture, found that the proportion of the population progressing through the cell cycle was different between genera, resulting in differing growth rates. Similarly, different Symbiodiniaceae species have been shown to have different proliferation rates and reach different densities within the same host^24–27^, with inherent differences in cell-cycle machinery between species being one possible explanation*.* The current study represents the first attempt to identify and describe cell-cycle proteins across diverse Symbiodiniaceae species and provides a basis for future research.

## Materials and methods

### Identification of Symbiodiniaceae CDKs and cyclins

Twenty-seven Symbiodiniaceae transcriptome and genome databases were acquired from publicly available sources (Table S2). Cyclins and CDKs from two free-living Symbiodiniaceae species, *Effrenium voratum*^28^ and *Fugacium kawagutii*^29^, were compared with symbiotic species (*Symbiodinium microadriaticum*, *S. tridacnidorum, Breviolum minutum, B. aenigmaticum, B. pseudominutum, B. psygmophilum, Cladocopium goreaui, Cladocopium* genotypes C15 and C92 and *Durusdinium trenchii*). Profile hidden Markov models (pHMMs) were obtained from the PFAM 31.0 database for the cyclin N terminal domain (PF00134) and CDK conserved domain (PF00069). The pHMM models were re-trained using CDKs and cyclins from eukaryotic organisms closely related to Symbiodiniaceae (e.g. Apicomplexa) using the aphid R package^30^.

The Symbiodiniaceae databases were then queried with the updated pHMM models using an optimal alignment homology search to find putative cyclin and CDK sequences (Fig. S1). Sequences with log-odds similarity scores > 50 were retained for cyclins and CDKs. The cyclin model returned 119 sequences and the CDK model returned 6032 sequences. Due to the high abundance of Symbiodiniaceae CDK sequences returned from the model, the collected CDK sequences from the pHMM model were examined further using conserved CDK motifs (Table S3)^31–34^. If the CDK contained a motif that when BLASTp searched against the NCBI non-redundant database matched to a CDK, the sequence was retained for further analysis. All 119 cyclins retrieved by the model were also searched, and were included in the analysis if the highest-scoring sequence was annotated as a cyclin or CDK and had an E value ≤ 1 × 10^−5^. Owing to the lack of information available for CDKs and cyclins in other unicellular marine eukaryotes, several taxa (Table S5) were chosen for screening through the trained pHMM models to identify putative cyclin and CDK sequences, allowing possible alveolate-specific groups to be identified.

### Sequence alignment and phylogenetic analysis

Phylogenetic trees were generated twice. The sequence alignment for the first set of trees was aligned to just the conserved cyclin N (PFAM ID:PF00134) and protein kinase domains (PFAM ID: PF00069), which were used to determine distinct phylogenetic groups of Symbiodiniaceae cyclins and CDKs. These were later used to identify other similar sequences from the Symbiodiniaceae databases.

The first trees were generated by aligning the putative CDKs and cyclins in the aphid R package^30^ (along with other eukaryotic cyclins and CDKs) and the best substitution model was selected by ProTest (v3.4)^35^. Both alignments had an appropriate evolutionary model of PROTOGAMMAAUTO, which was then used to infer maximum-likelihood trees in RAxML (v8.2.12)^36^. Bootstrap support was used to find the distinct phylogenetic groupings among Symbiodiniaceae CDKs and cyclins (*n* = 1000) by using the topology of the tree with the highest log-likelihood score. Trees were rooted using the *H. sapiens* MAPK (NP_002737.2) gene for the CDK tree and *H. sapiens* CABLES1 (NP_112492.2) and *H. sapiens* CABLES2 (NP_001094089.1) for the cyclin tree based on a previous study on animal cyclins and CDKs^37^. Symbiodiniaceae candidate proteins from distinct phylogenetic CDK and cyclin groups were used to perform custom BLASTp searches (Table S4) in Geneious v.11.1.5 (Biomatters Ltd.) against the 27 Symbiodiniaceae databases used in this study, to ensure that all putative CDKs and cyclins were identified. The first 10 Symbiodiniaceae proteins with the highest E-value (≤ 1 × 10^−5^) that were not previously identified by the pHMM model, and that identified a CDK or cyclin on the NCBI nr database in BLASTp searches, were collected from each Symbiodiniaceae database for each of the candidate proteins. These newly identified Symbiodiniaceae sequences were added to the previously collected sequences through the pHMM models and together these were entered into CD-Hit v4.8^38^ to remove isoforms and redundant proteins using a similarity threshold of 90%.

Once redundant proteins and isoforms were removed, Symbiodiniaceae sequences were submitted to InterProScan^39^ to identify CDK and cyclin domains. Due to the low-quality annotations in Symbiodiniaceae databases^40^, many sequences contained regions that coded other proteins, therefore the alignments were trimmed manually in Geneious v.11.1.5 to CDK- (PFAM ID: PF00069; PANTHER ID: PTHR24056) and cyclin-(PFAM ID:PF00134, PF02984, PF16899 and PF08613; PANTHER ID: PTHR10177) annotated domains. The final CDK alignment for the second phylogenetic analysis was 465 amino acids (aa) long, and contained 177 Symbiodiniaceae sequences and 50 CDKs from other eukaryotes (Supplementary File S1), whereas the cyclin alignment was 395 aa long and contained 191 Symbiodiniaceae sequences and 54 cyclins from other eukaryotes (Supplementary File S2). All CDK and cyclin families from *Homo sapiens* were included in the trees to create the correct topologies, and CDKs and cyclins from other model organisms, including *Saccharomyces cerevisiae* and *Arabidopsis thaliana,* were only included if Symbiodiniaceae proteins were related to them.

Final CDK and cyclin alignments were run through ProTest (v3.4)^35^ as described previously. Maximum-likelihood trees were then run in PhyML (v3.1)^41^ using the Akaike information criterion, which corresponded to the LG + I + G + F model for the CDK alignment with a proportion of invariable sites of 0.039 and a gamma shape parameter of 1.195, and the LG + G + F model for cyclin alignments with a gamma shape parameter of 2.331. Due to the quantity of sequences in the tree, an approximate likelihood ratio test (aLRT) was used for branch support instead of bootstrap support^42^, however it has been shown to be very similar in calculating correct branch supports^43^. Based on a comparison of correct branch topologies determined by bootstrap support and SH-values^43^, true Symbiodiniaceae CDK and cyclin homologs were determined by branches containing an SH-value > 0.8. Trees were rooted as described previously. Trees were edited in the Interactive Tree of Life (iToL) software v.5.6.3^44^. The nomenclature of protein groups that did not phylogenetically group with other well-classified CDKs or cyclins was attributed by using BLAST searches against the NCBI nr database.

### Cyclin and CDK gene expression of *Breviolum minutum*

To explore expression of cyclins and CDKs in Symbiodiniaceae, RNA-Seq reads were analysed from a recent study by Maor‐Landaw et al*.*^45^ on the expression of cultured (*n* = 3) and freshly isolated *Breviolum minutum* (*n* = 3) from the sea anemone *Exaiptasia diaphana* (= *pallida*) (SRA PRJNA544863). Reads were aligned to the *B. minutum* genome assembly^46^ using STAR v2.7.1a in two-pass mode^47^ and read counts were extracted from the alignments with featureCounts v1.6.3^48^. Differential expression analysis was completed using the exact test in EdgeR^49^ on TMM normalized counts of the cultured and isolated *B. minutum*. Differentially expressed genes (DEGs) were those with Benjamini–Hochberg adjusted *p*-values < 0.05. Cyclins and CDKs identified in *B. minutum* were selected from the list of DEGs to generate a heat map in the R environment^50^, using the mean–variance modelling at the observational level (voom)^51^ of log_2_-transformed counts *per* million (CPM).

## Results and discussion

### Characterisation and phylogenetic positioning of Symbiodiniaceae CDK sequences

**Figure 2:**
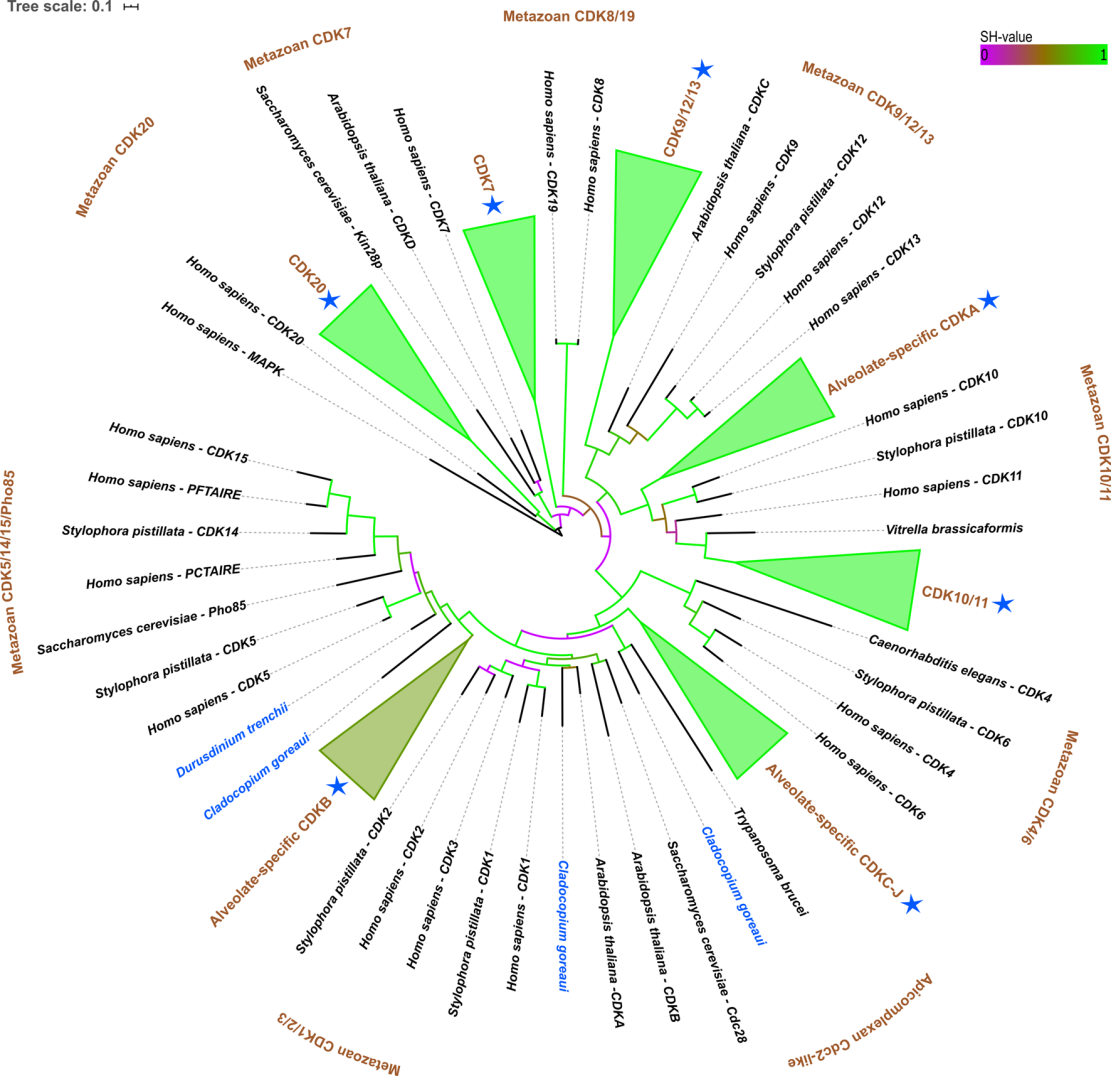
Collapsed phylogenetic tree of CDKs in the Symbiodiniaceae. Colour of branches corresponds to aLRT support (SH-value). Purple branches correspond to SH-values below 0.5, brown branches correspond to SH-values near 0.5, and green branches correspond to SH-values close to 1. Symbiodiniaceae species are written in blue, and blue stars depict collapsed branches containing Symbiodiniaceae species. The tree was made using PhyML(v3.1)^41^ and visualised using the Interactive Tree of Life software (v5.6.3)^44^.

Eukaryotic organisms contain different numbers of CDK proteins, ranging from three in premetazoans, to 20 in eumetazoans such as *Homo sapiens*^37^. A total of 177 unique Symbiodiniaceae CDK gene copies were identified across six genera (Table 1). CDK gene copy numbers were the highest in *Cladocopium goreaui*, which contained 16 CDK copies*.* Interestingly, no CDKs related to the CDK4/6 family nor their cyclin partners (cyclin E) were found in Symbiodiniaceae using the databases referenced in this study (Table 1; Fig. 2). This agrees with findings for plants and many protists, in which there is also an absence of the CDK4/6 family and cyclin E in most pre-metazoan lineages^37^.

**Table 1:** Gene copies of CDKs in the Symbiodiniaceae.

	Source of database	CDK1/2/3 subfamily	CDK4/6 subfamily	CDK5 subfamily (CDK5/14/15/Pho85)	CDK7	CDK8 subfamily (CDK8/19)	CDK9 subfamily (CDK9/12/13)	CDK10/CDK11 subfamily	CDK20	Alveolate-specific CDKA	Alveolate-specific CDKB	Alveolate-specific CDKC	Alveolate-specific CDKD	Alveolate-specific CDKE	Alveolate-specific CDKF	Alveolate-specific CDKG	Alveolate-specific CDKH	Alveolate-specific CDKI	Alveolate-specific CDKJ	Parasitic CDKA	Apicomplexan Cdc2-like CDK	Total
*S. microadriaticum*	G				1				1	1	1	1	1		1	1	1	1	1			11
*S. tridacnidorum*	G				1		1	1		1	1	1	1	1	1		1	1	1			12
*Symbiodinium* sp. #1	T				1		1	1			1	1	1	1	1	1		1				10
*Symbiodinium* sp. #2	T				1			1			1			1				1				5
*B. minutum*	G				1		1	1	1	1	1	1	1	1	1	1	1	1	1			14
*B. aenigmaticum*	T				1		1	1			1	1	1	1	1		1	1	1			11
*B. pseudominutum*	T				1		1	1		1	1	1	1	1	1	1		1	2			13
*B. psygmophilum*	T				1		1	1		1	1	1	1	1	1	1	1	1	1			13
*Breviolum* sp. #1	T				1		1	1		1	1	1	1	1	1		1	1	2			13
*C. goreaui*	G	1		1	1		1	1		1	1	1	1	1	1	1		1	2		1	16
*Cladocopium* sp. C15	T				1						1	1	1	1	1		1	1	1			9
*Cladocopium* sp. C92	G						1		1		1	1	1	1	1	1	1		1			10
*Cladocopium* sp. #1	T										1			1						1		3
*Cladocopium* sp. #2	T				1		1	1			1	1	1	1	1		1	1				10
*Cladocopium* sp. #3	T										1	1		1								2
*D. trenchii*	T			1							1	1	1	1						1		6
*Durusdinium* sp. #1	T									1		1		1				1	1			4
*Effrenium voratum*	T										1	1	1	1	1			1	1			7
*Fugacium kawagutii*	G				1			1	1	1	1	1										5
TOTAL		1	0	2	13	0	10	11	4	9	18	17	14	17	13	7	9	14	15	2	1	177

**Figure 3:**
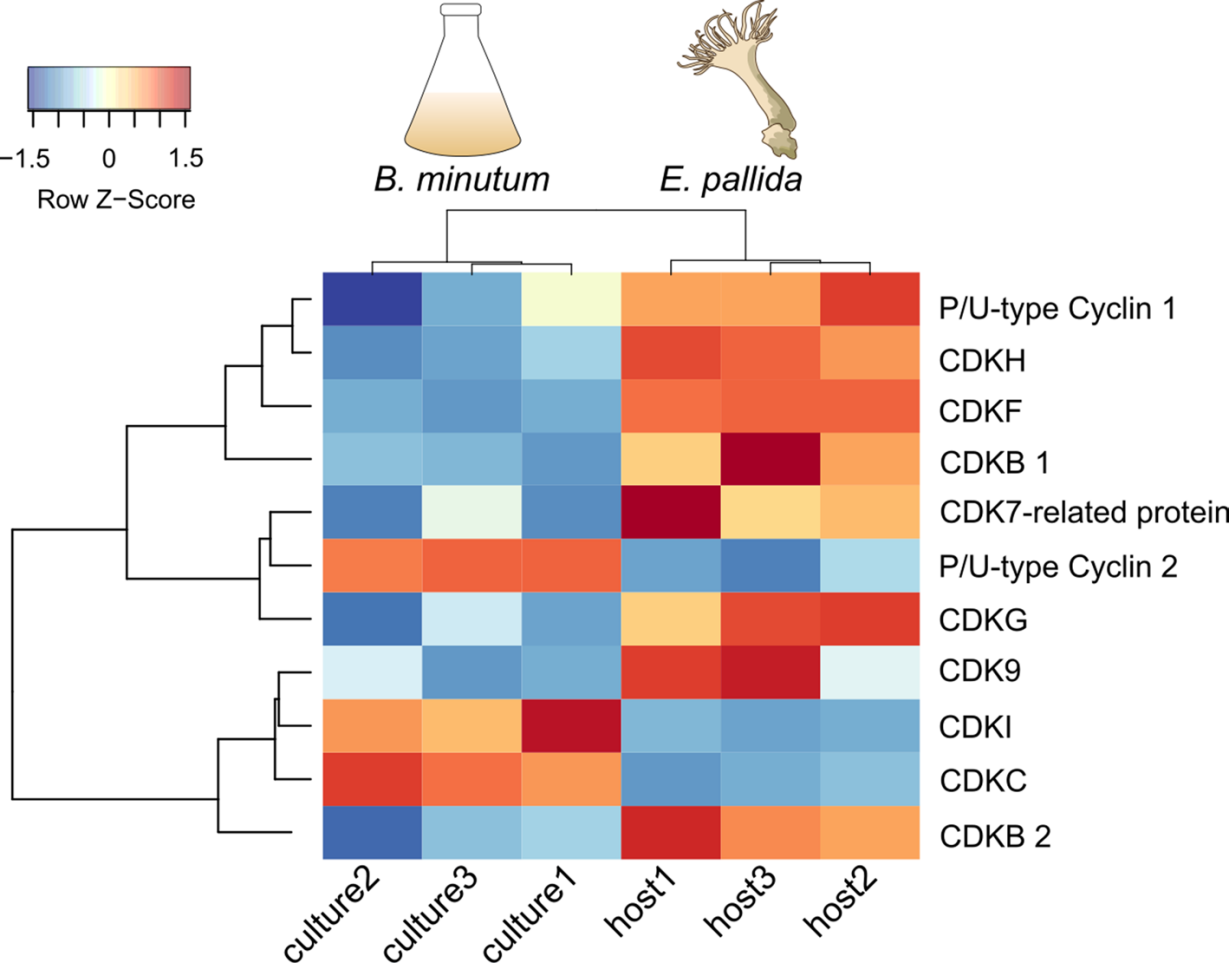
Heat map comparison of *Breviolum minutum* cyclin and CDK gene expression between culture and cells freshly isolated from *Exaiptasia pallida*. Red corresponds to a higher Z-score and gene up-regulation whilst blue corresponds to a lower Z-score and down-regulation.

Some of the Symbiodiniaceae CDKs showed high sequence similarity to eumetazoan CDKs, however the largest expansion of CDKs was within the alveolate-specific CDK groups (Table 1, Fig. 2). A previous study^20^ investigating Symbiodiniaceae cell-cycle proteins found four *B. minutum*-specific CDKs*.* Here we show that three of those four CDKs are also present across other Symbiodiniaceae species (alveolate-specific CDKG/H/J—Table 1; Supplementary Fig. S2). In the previous study^20^, the *B. minutum* CDKs (alveolate-specific CDKG/H/J) did not change their expression with cell-cycle phase when in a free-living state. However, our analysis of the previously published RNA-Seq data^45^ shows that symbiosis alters the expression of *B. minutum* CDKG and CDKH, which were both up-regulated *in hospite* compared to when in culture (Table S6; Fig. 3).

The most common CDK identified in Symbiodiniaceae was an alveolate-specific CDK (CDKB) with gene copies found across 18 species in the five Symbiodiniaceae genera examined (Table 1). Symbiodiniaceae proteins in the CDKB group contained the canonical CDK motif PSTAIRE (Table 2). The CDKB sister clade is the Pho85/CDK5 subfamily (SH-value 0.95), which is sister to the metazoan CDK1/*S. cerevisae* Cdc28, with strong branch support (SH-value = 1; Supplementary Fig. S2). CDK1/Cdc28 is the primary cell-cycle regulator from yeast to humans^52–54^, however Pho85 has been shown to have overlapping roles with Cdc28, phosphorylating many of the same substrates^55^. The primary roles of Pho85 include responding to environmental cues via the induction of signals that inform the cell whether conditions are adequate for cell division and nutrient metabolism ^56^. As Symbiodiniaceae proliferate in response to increased nutrients^5^, they may have evolved CDKs that possess similar functions for linking external stimuli (e.g. environmental nitrogen and phosphorus levels) to cell-cycle progression. Furthermore, our analysis of the RNA-Seq data comparing cultured *versus* freshly-isolated *B. minutum*^45^ suggests that two CDKB genes are up-regulated in symbiosis (Table S6; Fig. 3). We hypothesise that, due to its phylogenetic grouping, conserved motif, widespread presence across Symbiodiniaceae and up-regulation in the symbiotic state, CDKB may be a homolog of Cdc28/Pho85 and a primary cell-cycle regulator in Symbiodiniaceae. This hypothesis requires confirmation.

**Table 2:** Conserved motifs found in the Symbiodiniaceae CDK genes.

CDK group	Symbiodiniaceae motif
CDK1/2/3 subfamily	PSTALRE
CDK4/6 subfamily	N/A
CDK5 subfamily (CDK5/14/15/Pho85)	PCTAIRE
CDK7	(G/S)TALRE
CDK8 subfamily (CDK8/19)	N/A
CDK9/12/13	P(A/T/S)T(S/A/C)(I/V)RE
CDK10/11	P(V/S)(P/A/S)S(L/I)RE
CDK20	PWFSAERE
Alveolate-specific CDKA	P(K/R)(I/S)SLRE
Alveolate-specific CDKB	PSTAIRE
Alveolate-specific CDKC	PSTAIRE
Alveolate-specific CDKD	PSTALRE/EHQLRRE
Alveolate-specific CDKE	P(G/S)TA(I/L)RE
Alveolate-specific CDKF	(S/P)(A/P)(T/H/Y/Q)(T/A/V)(I/L)RE
Alveolate-specific CDKG	S(A/T)Q(V/A)LRE
Alveolate-specific CDKH	(S/T)S(Y/F)(S/A)(L/I)RE
Alveolate-specific CDKI	P(T/A)(T/A)(S/T/A)(I/L)RE
Alveolate-specific CDKJ	P(T/A)TALRE; PAVA(L/M)RE
Parasite CDKA	PSTAIRE
Apicomplexan Cdc2-like CDK	PQTALRE

Proteins related to eumetazoan transcriptional CDK subfamilies (CDK9/12/13 (SH-value = 0.89), CDK10/11 (SH-value = 0.89) and CDK20 (SH-value = 0.93)) were also present in Symbiodiniaceae (Table 1; Supplementary Fig. S2). Amongst transcriptional roles, the CDK10/11 subfamily has also been proposed to have roles in cell-cycle progression during the G_2_/M phase (Table S1)^57^. However, in *B. minutum,* CDK20, CDK9 and CDK11 expression did not change with cell-cycle phase^20^, highlighting their similarity to metazoan CDK20, CDK9 and CDK11, which are predominantly transcriptional CDKs and indirectly related to the cell cycle^58^. Previous studies^20^ have reported an absence of CDK7 in *B. minutum*, however this study found a CDK7-related gene (confirmed via BLAST searches on the NCBI nr database) across 13 different Symbiodiniaceae species (Supplementary Fig. S2). The difference in results may be explained, in part, by the Symbiodiniaceae CDK7 being phylogenetically distant from the metazoan CDK7 and yeast CDK7 homolog (Kin28p), grouping separately and with no concrete relationship to any other CDK included in this study, possibly owing to its divergence. CDK7 has been discovered in other basal organisms, such as the amoebozoan *Dictyostelium purpureum*^59^. In metazoans, CDK7 forms part of the cyclin kinase-activating (CAK) complex that activates other CDKs by phosphorylating their T-loop^60^, and inhibition of CDK7 led to the arrest of the cell cycle in proliferating cells^61^. The previously published RNA-Seq data^45^ show that the CDK7-related gene was up-regulated in symbiotic *B. minutum* (Table S6).

*Symbiodinium* sp. #2 contained CDKs and cyclins that are more similar to those of the free-living dinoflagellate *Amphidinium* (SH-value > 0.95) than other Symbiodiniaceae species (Supplementary Fig. S2). CDKs and cyclins that are not present in *Amphidinium* sp. but are present in *Symbiodinium* sp. #2 grouped next to, not with, the other Symbiodiniaceae species (SH-value > 0.78). This placement may reflect the basal status of *Symbiodinium* within the Symbiodiniaceae^21^.

Several Symbiodiniaceae species contained CDKs found in parasitic taxa. A CDK protein that is related to a gene present in the free-living, facultative pathogenic marine ciliate *Pseudocohnilembus persalinus,* was found in both *D. trenchii* and *Cladocopium* sp. #1 (SH-value = 1), while *C. goreaui* harbours a CDK related to Cdc2-related kinase 6 (CRK6) from *Trypanosoma brucei* (SH-value = 0.97) (Fig. 2, Supplementary Fig. S2). Studies^62,63^ have shown that the loss of *T. brucei* CRK6 slows cell growth but does not inhibit the cell cycle (contrasting with cell cycle indispensable CRK3 and CRK1), highlighting a function of CRK6 that may not be directly associated with the cell cycle.

### Characterisation and phylogenetic positioning of Symbiodiniaceae cyclin sequences

**Figure 4:**
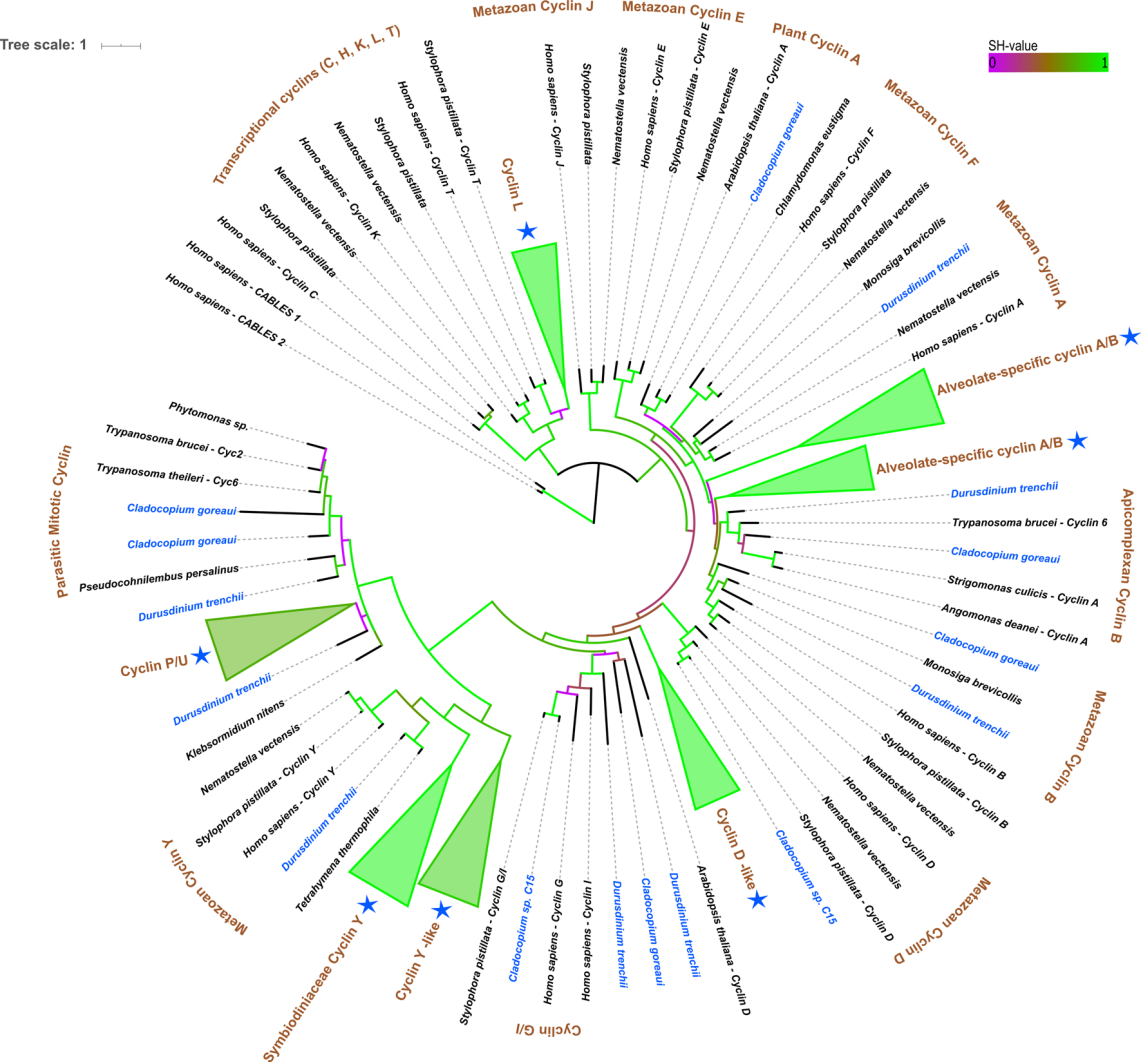
Collapsed phylogenetic tree of cyclins in the Symbiodiniaceae. Colour of branches corresponds to aLRT support (SH-value). Purple branches correspond to SH-values below 0.5, brown branches correspond to SH-values near 0.5, and green branches correspond to SH-values close to 1. Symbiodiniaceae species are written in blue, and blue stars depict collapsed branches containing Symbiodiniaceae species. The tree was made using PhyML(v3.1)^41^ and visualised using the Interactive Tree of Life software (v.5.6.3)^44^.

Similar to CDKs, the number of cyclins differs across eukaryotes – from eight in premetazoans to 29 in *Homo sapiens*^37^. Across the six Symbiodiniaceae genera examined, 191 cyclins were identified (Table 3; Fig. 4). *C. goreaui* contained the most cyclin gene copies, harbouring 19 distinct copies. Differences in abundance of cell-cycle proteins (cyclins and CDKs) between different Symbiodiniaceae species could be a result of the different database information provided (genomes *versus* transcriptomes), as if CDKs and cyclins were not expressed at the time of transcriptomic analysis, these may have been missed, thus producing a bias towards genomes harbouring more cyclin and CDK gene copies. Another possible reason for the difference in cyclin and CDK gene copies in the Symbiodiniaceae are gene duplication events, which are followed by genetic drift over time, causing the formation of cell-cycle paralogs with functional divergence in the family.

**Table 3:** Gene copies of cyclins in the Symbiodiniaceae and complementary conserved motifs.

	Cyclin type	Symbiodiniaceae motif	*Symbiodinium microadriaticum*	*Symbiodinium triacnidorum*	*Symbiodinium* sp.#1	*Symbiodinium* sp. #2	*Breviolum minutum*	*Breviolum aenigmaticum*	*Breviolum pseudominutum*	*Breviolum psygmophilum*	*Breviolum* sp. #1	*Cladocopium goreaui*	*Cladocopium* sp. C15	*Cladocopium* sp. C92	*Cladocopium* sp. #1	*Cladocopium* sp.#2	*Cladocopium* sp. #3	*Durusdinium trenchii*	*Durusdinium* sp.#1	*Effrenium voratum*	*Fungacium kawagutii*	Total
Eumetazoan and plant cell-cycle cyclins	Cyclin A	(M/L)R(A/V)(I/A)L(V/I)DWL										1						1				2
	Cyclin B	YRTKIVNWM; NLAVLHDWL										1						1				2
	Cyclin D	MRRMVTSWM											1									1
	Plant Cyclin D-like	ERALAVDWL; DRQETLTWM; RRLDALEWL	1	1	1		2	2	2	2	1	2	2	2				1		1		20
	Cyclin E																					0
	Cyclin G/I	GRRDLMIWL;QRDNITTFM;(W/N)R(R/D)(Q/D)(M/S)(I/T)(E/V)(W/F)(C/I)										1	1					2				4
	Cyclin J/O																					0
	Cyclin F																					0
Eumetazoan transcriptional cyclins	Cyclin C																					0
	Cyclin H																					0
	Cyclin K																					0
	Cyclin L	LR(R/A)FG(V/G/N/S)VL(I/L)	2	1	1	1	2		2	2	2	2	1	2								18
	Cyclin T																					0
	Cyclin Y-like	LADEIYELL; S(K/T)E(T/A)ILDFL; REMVLDFL; HEAVL(T/A)FL		2	2		1		1	1	1	1		1		1		1	1	2		15
	Cyclin Y	TVDNIYEFM; –IYDFL	1	1		1	1				1	1								1		7
Protist A/B cyclins	Cyclin A/B	MR(G/A)ILVDWL; ER(A/G)(L/T/A/S/C/I)(A/V)(A/D/N)W(L/M); (S/Q)RA(V/T)(Q/L)(I/V)D(F/M)(M/I)	2	4	3	2	3	4	3	3	3	3	3	3	2	4	1	2	2	5	2	54
	Apicomplexan cyclin B	MR(T/I)ILVDWL										1						1				2
	Parasitic mitotic cyclin	PSINVADYL; PGITMPDFF; PPLSLADLG										2						1				3
	P/U cyclin	PSISVRSYL; PPIT(V/L)(R/K)DY(V/L); (E/D)PPDI(S/N)(A/Y/S)(Y/F)(I/V); (K/S)(N/A)MDLDDFI; E(S/T)(S/Q/V)DIEEYI; P(T/S)I(S/G)(V/I)(G/E)(E/D)YL; PKISV(R/L)(D/N)YL; PGIG(V/A/I)(A/E)(A/Q/L/V)YL; P(G/T/S)I(P/S)V(D/Q)(K/Q)YL	5	5	4		4	4	4	4	4	4	2	3	2	6	1	2	1	6	2	63
Total			11	14	11	4	13	10	12	12	12	19	10	11	4	11	2	12	4	15	4	191

**Figure 5:**
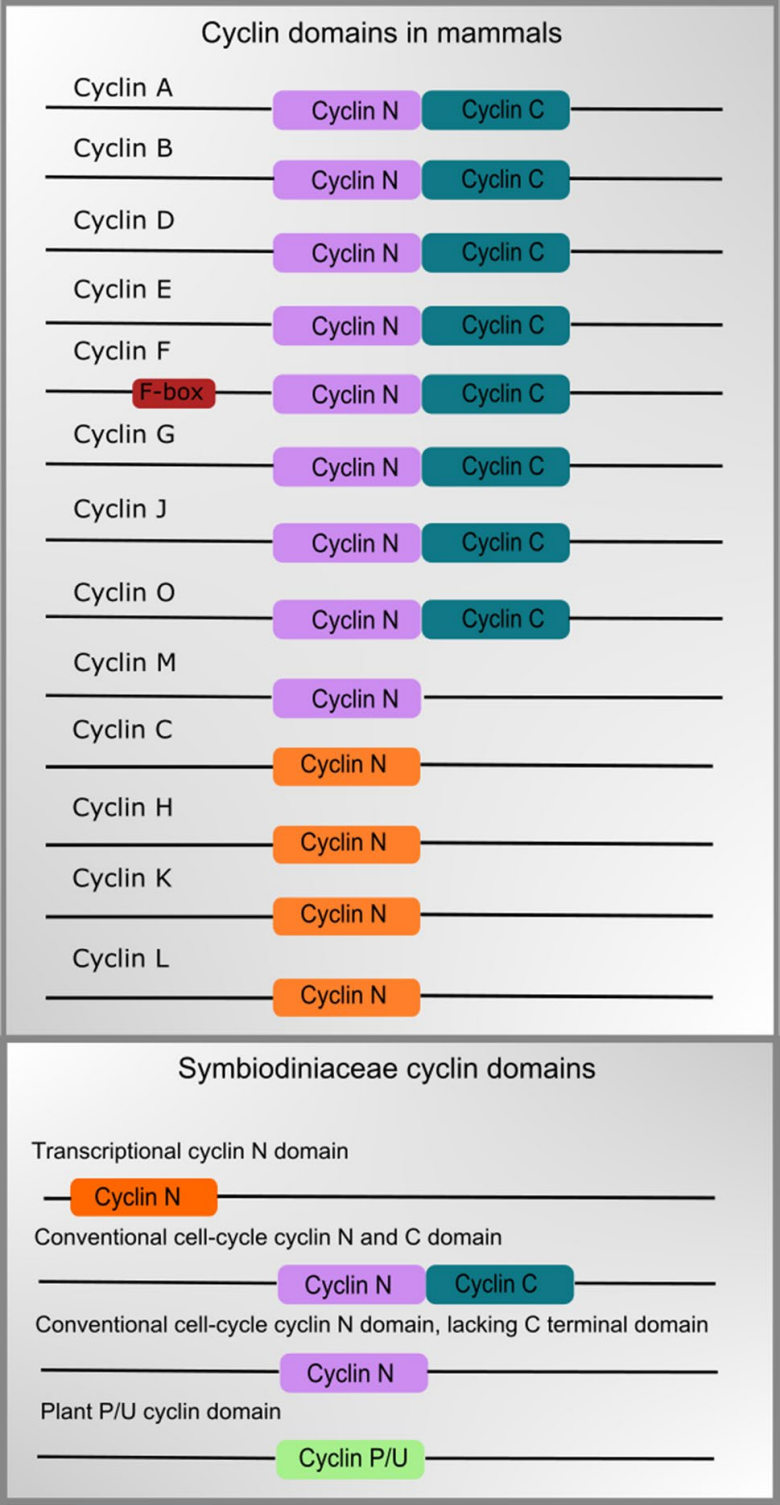
Domain structure of cyclin proteins present in the Symbiodiniaceae.

All the cyclins found in the Symbiodiniaceae contained one of three distinct domains (Fig. 5): the conventional cell-cycle cyclin N and C domains; a cyclin N domain found nearer the amino terminus than the position of the conventional cell-cycle cyclin N domain which corresponded phylogenetically to transcriptional cyclins (specifically cyclin L); and a single plant P/U cyclin domain that is phylogenetically related to the analogous domain of the Pho80p cyclin in *S. cerevisiae*.

Proteins related to eukaryotic cell-cycle cyclins A, B, D and G/I, and transcriptional cyclin L were identified in the Symbiodiniaceae, along with proteins related to plant cyclin D, protist/plant P/U-type cyclin and cyclin Y, as well as genes related to Cyc2 and mitotic Cyc6 from the sister taxon Apicomplexa (Fig. 4; Supplementary Fig. S3). Three phylogenetically distinct groups of cyclins were also present in Symbiodiniaceae, that upon searching the NCBI nr database, matched to alveolate-specific cyclins A/B (Supplementary Fig. S3). Two cyclins previously reported to be *B. minutum*-specific^20^ were found in other Symbiodiniaceae species and belong to the “Plant Cyclin D-like” grouping (Table 3; Supplementary Fig. S3). In metazoans and plants, cyclin D is required for G_1_ phase progression^64^.

An expansion of the protist/plant P/U-type cyclin groups was found within Symbiodiniaceae, with 63 gene copies being present across six Symbiodiniaceae genera (Table 3, Fig. 4, Supplementary Fig. S3). This finding agrees with the previous study^20^, which found P-type cyclins in *B. minutum.* Genes within these groups were related to the *S. cerevisiae* Pho80p cyclin. In *S. cerevisiae,* the Pho80 subfamily of P/U-type cyclins (Pho80, Pcl6, Pcl7, Pcl8 and Pcl10^55^) links nutrient availability with cell-cycle progression^65^. In *A. thaliana,* P/U-type cyclins are implicated in the switch from heterotrophic to autotrophic growth^66^. RNA-Seq data^45^ revealed that two of these P/U type cyclins had contrasting expression (one being up-regulated whilst the other was down-regulated) *in hospite versus* in culture in *B. minutum* (Table S6; Fig. 3). Given that nutritional exchange is a fundamental feature of the cnidarian–dinoflagellate symbiosis^1^, and that P/U cyclins are involved in glycogen metabolism and carbon source utilisation^56,67^, the differential expression of these cyclins *in hospite* is unsurprising. Whether the difference in expression is a response to environmental stimuli exclusively experienced in symbiosis, e.g. host-associated factors such as the pH of the symbiosome in which the alga resides^68^, requires further study. Similar to Symbiodiniaceae, the apicomplexan *T. gondii* also lacks a cyclin E homolog and instead uses a P-type cyclin for G_1_ phase progression^69^. Symbiodiniaceae may also use P-type cyclins in place of eumetazoan cyclin E, however this requires confirmation.

Twenty two cyclin Y-like gene copies were found across the Symbiodiniaceae. These encompassed two phylogenetic groups, one termed “Cyclin Y” which grouped with eumetazoan Cyclin Y (SH-value = 0.93), and one group of cyclins that grouped as a sister group with the conventional eumetazoan Cyclin Y (SH-value = 0.80) that were termed “Cyclin Y-like” (Fig. 4, Supplementary Fig. S3). Cyclin Y is absent in plants and fungi (being replaced by the Pcl class of cyclins in fungi) but is present in animals and protists^59^. In eumetazoans and fungi, cyclin Y and Pcl1 cyclins are the binding partners of CDK14 and Pho85, respectively^70,71^. In yeast, the cyclin Y homolog, Pcl1, is expressed during the G_1_ phase of the cell cycle^70^ and provides information to the cell, determining whether it passes the START checkpoint, where the yeast cell commits to mitosis^56^. In *Drosophila*, cyclin Y is required for Wnt signalling by localising the CDK14 kinase to the cell membrane^72^. As Wnt signalling is an indispensable pathway for the long-term viability of cells^73^, the presence of cyclin Y and cyclin-Y like genes in most eukaryotes is predicted.

Uniquely, *C. goreaui* and *D. trenchii* both contain cyclins present in two phylogenetic groups that cluster with mitotic cyclins from the dinoflagellate sister taxon, the apicomplexans^74^ (Fig. 4). One group is related to the B-type G_2_/M phase-specific cyclin, Cyc6, in the apicomplexans (SH-value > 0.98)*,* while the other clusters with Cyc2-like from *T. brucei*, which is involved in transition from both the G_1_ to S and G_2_ to M phases^75^ (Fig. 4). The correlation in cell-cycle machinery of both cyclins and CDKs between pathogenic protists and *D. trenchii,* which is reported to colonise hosts during heat stress opportunistically^25,76^ and has a fast growth rate *versus* other Symbiodiniaceae species in culture^23^, is noteworthy and warrants future investigation.

*Cladocopium* sp. C15 harbours two cyclins (cyclin D and cyclin G/I) that are related to those in the symbiotic coral, *Stylophora pistillata*, with strong support (SH-value = 1). Both *Cladocopium* sp. C15 cyclin D and G/I share a similar identity (92.1% and 74.5%, respectively) and similarity (95.7% and 91.6%, respectively), across the full sequence length to *S. pistillata* cyclins. To account for possible contamination of host material in the *Cladocopium* sp. C15 transcriptome, the origin of this symbiont was traced^77^. The *Cladocopium* sp. C15 was found to have been freshly isolated from its host *Porites compressa,* so host contamination cannot be excluded. This being said, symbiosis has been suggested to drive the formation of paralogous genes involved in host-symbiont interactions due to selective pressure for a more mutualistic partnership between host and symbiont^78^. How the evolution of cell-cycle proteins that share a high similarity between host and symbiont affects biomass co-ordination is deserving of future attention.

## Conclusions

Our study shows the divergence of cell-cycle proteins in the Symbiodiniaceae family and demonstrates that there are several conserved CDK and cyclin groups across the Symbiodiniaceae, though also marked species-specific differences. Which of these conserved cell-cycle proteins are indispensable for cell-cycle progression and which species-specific proteins influence proliferation rates in symbiosis remains unknown. Further study will be required to clarify which CDKs and cyclins are required for Symbiodiniaceae cell-cycle progression, and whether this differs between species and symbiotic states. As annotation of Symbiodiniaceae genomes is challenging^79^, future studies should aim to apply the same comparative analysis across new Symbiodiniaceae genomes to inform cyclin and CDK gene prediction accurately.

## Supplementary information

Supplementary Information 1.

Supplementary File 1.

Supplementary File 2.

Supplementary Information 2.

Supplementary Information 2.

Supplementary Figure 2.

Supplementary Figure 3.

## References

[1] Davy SK, Allemand D, Weis VM (2012). Cell biology of cnidarian–dinoflagellate symbiosis. Microbiol. Mol. Biol. Rev..

[2] Wakefield TS, Kempf SC (2001). Development of host-and symbiont-specific monoclonal antibodies and confirmation of the origin of the symbiosome membrane in a cnidarian–dinoflagellate symbiosis. Biol. Bull..

[3] Jones RJ, Yellowlees D (1997). Regulation and control of intracellular algae (= zooxanthellae) in hard corals. Philos. Trans. R. Soc. Lond. B..

[4] Hoegh-Guldberg O, Smith GJ (1989). Influence of the population density of zooxanthellae and supply of ammonium on the biomass and metabolic characteristics of the reef corals *Seriatopora hystrix* and *Stylophora pistillata*. Mar. Ecol. Prog. Ser..

[5] Hoegh-Guldberg O (1994). The population dynamics of symbiotic zooxanthellae in the coral *Pocillopora damicornis* exposed to elevated ammonium (NH _4_ Cl) concentrations. Pac. Sci..

[6] Muller-Parker G, McCloskey LR, Hoegh-Guldberg O, McAuley PJ (1994). Effect of ammonium enrichment on animal and algal biomass of the coral *Pocillopora damicornis*. Pac. Sci..

[7] Smith GJ, Muscatine L (1999). Cell cycle of symbiotic dinoflagellates: variation in G_1_ phase-duration with anemone nutritional status and macronutrient supply in the *Aiptasia pulchella*–*Symbiodinium pulchrorum* symbiosis. Mar. Biol..

[8] Fitt WK (2000). Cellular growth of host and symbiont in a cnidarian–zooxanthellar symbiosis. Biol. Bull..

[9] Xiang T (2020). Symbiont population control by host-symbiont metabolic interaction in Symbiodiniaceae–cnidarian associations. Nat. Commun..

[10] Baghdasarian G, Muscatine L (2000). Preferential expulsion of dividing algal cells as a mechanism for regulating algal–cnidarian symbiosis. Biol. Bull..

[11] Dunn SR, Schnitzler CE, Weis VM (2007). Apoptosis and autophagy as mechanisms of dinoflagellate symbiont release during cnidarian bleaching: every which way you lose. Proc. R. Soc. Lond. B.

[12] Pardee AB (1989). G_1_ events and regulation of cell proliferation. Science.

[13] Nishitani H, Lygerou Z (2002). Control of DNA replication licensing in a cell cycle. Genes Cells.

[14] Stark GR, Taylor WR (2004). Analyzing the G_2_/M checkpoint. Methods Mol. Biol..

[15] Houtgraaf JH, Versmissen J, van der Giessen WJ (2006). A concise review of DNA damage checkpoints and repair in mammalian cells. Cardiovasc. Revascularization Med..

[16] Barnum KJ, O’Connell MJ (2014). Cell-cycle regulation by checkpoints. Methods Mol. Biol..

[17] Malumbres M, Barbacid M (2009). Cell cycle, CDKs and cancer: a changing paradigm. Nat. Rev. Cancer.

[18] Lim S, Kaldis P (2013). CDKs, cyclins and CKIs: roles beyond cell cycle regulation. Development.

[19] Vermeulen K, Van Bockstaele DR, Berneman ZN (2003). The cell cycle: a review of regulation, deregulation and therapeutic targets in cancer. Cell Prolif..

[20] Cato ML (2019). Genome-wide analysis of cell cycle-regulating genes in the symbiotic dinoflagellate *Breviolum minutum*. Genes Genomes Genet..

[21] LaJeunesse TC (2018). Systematic revision of Symbiodiniaceae highlights the antiquity and diversity of coral endosymbionts. Curr. Biol..

[22] Nitschke MR (2020). Description of *Freudenthalidium* gen. nov. and *Halluxium* gen. nov. to formally recognize clades Fr3 and H as Genera in the Family Symbiodiniaceae (Dinophyceae). J. Phycol..

[23] Fujise L (2018). Cell cycle dynamics of cultured coral endosymbiotic microalgae (*Symbiodinium*) across different types (species) under alternate light and temperature conditions. J. Eukaryot. Microbiol..

[24] Yuyama I, Higuchi T (2014). Comparing the effects of symbiotic algae (*Symbiodinium*) clades C1 and D on early growth stages of *Acropora tenuis*. PLoS ONE.

[25] Leal MC (2015). Symbiont type influences trophic plasticity of a model cnidarian–dinoflagellate symbiosis. J. Exp. Biol..

[26] Starzak DE, Quinnell RG, Nitschke MR, Davy SK (2014). The influence of symbiont type on photosynthetic carbon flux in a model cnidarian–dinoflagellate symbiosis. Mar. Biol..

[27] Gabay Y, Weis VM, Davy SK (2018). Symbiont identity influences patterns of symbiosis establishment, host growth, and asexual reproduction in a model cnidarian–dinoflagellate symbiosis. Biol. Bull..

[28] Jeong HJ (2014). Genetics and morphology characterize the dinoflagellate *Symbiodinium voratum*, n. sp., (Dinophyceae) as the sole representative of *Symbiodinium *clade E. J. Eukaryot. Microbiol..

[29] Liu H (2018). *Symbiodinium* genomes reveal adaptive evolution of functions related to coral–dinoflagellate symbiosis. Commun. Biol..

[30] Wilkinson SP (2019). aphid: an R package for analysis with profile hidden Markov models. Bioinformatics.

[31] Joubes J (2000). CDK-related protein kinases in plants. Plant Mol. Biol..

[32] Corellou F, Camasses A, Ligat L, Peaucellier G, Bouget F-Y (2005). Atypical regulation of a green lineage-specific B-type cyclin-dependent kinase. Plant Physiol..

[33] Malumbres M, Barbacid M (2005). Mammalian cyclin-dependent kinases. Trends Biochem. Sci..

[34] Talevich E, Mirza A, Kannan N (2011). Structural and evolutionary divergence of eukaryotic protein kinases in Apicomplexa. BMC Evol. Biol..

[35] Darriba D, Taboada GL, Doallo R, Posada D (2011). ProtTest 3: fast selection of best-fit models of protein evolution. Bioinformatics.

[36] Stamatakis A (2014). RAxML version 8: a tool for phylogenetic analysis and post-analysis of large phylogenies. Bioinformatics.

[37] Cao L (2014). Phylogenetic analysis of CDK and cyclin proteins in premetazoan lineages. BMC Evol. Biol..

[38] Li W, Jaroszewski L, Godzik A (2001). Clustering of highly homologous sequences to reduce the size of large protein databases. Bioinformatics.

[39] Quevillon E (2005). InterProScan: protein domains identifier. Nucleic Acids Res..

[40] Chen Y, González-Pech RA, Stephens TG, Bhattacharya D, Chan CX (2020). Evidence that inconsistent gene prediction can mislead analysis of dinoflagellate genomes. J. Phycol..

[41] Guindon S, Gascuel O (2003). A simple, fast, and accurate algorithm to estimate large phylogenies by maximum likelihood. Syst. Biol..

[42] Anisimova M, Gascuel O (2006). Approximate likelihood-ratio test for branches: a fast, accurate, and powerful alternative. Syst. Biol..

[43] Guindon S (2010). New algorithms and methods to estimate maximum-likelihood phylogenies: assessing the performance of PhyML 3.0. Syst. Biol..

[44] Letunic I, Bork P (2019). Interactive tree of life (iTOL) v4: recent updates and new developments. Nucleic Acids Res..

[45] Maor-Landaw K, van Oppen MJH, McFadden GI (2020). Symbiotic lifestyle triggers drastic changes in the gene expression of the algal endosymbiont *Breviolum minutum* (Symbiodiniaceae). Ecol. Evol..

[46] Shoguchi E (2013). Draft assembly of the *Symbiodinium minutum* nuclear genome reveals dinoflagellate gene structure. Curr. Biol..

[47] Dobin A (2013). STAR: ultrafast universal RNA-seq aligner. Bioinformatics.

[48] Liao Y, Smyth GK, Shi W (2014). featureCounts: an efficient general purpose program for assigning sequence reads to genomic features. Bioinformatics.

[49] Robinson MD, McCarthy DJ, Smyth GK (2010). edgeR: a Bioconductor package for differential expression analysis of digital gene expression data. Bioinformatics.

[50] Team, R. C. R: A Language and Environment for Statistical Computing. Vienna: R Project. Available at: https://www.R-project.org (2020).

[51] Law CW, Chen Y, Shi W, Smyth GK (2014). voom: precision weights unlock linear model analysis tools for RNA-seq read counts. Genome Biol..

[52] Santamaría D (2007). CDK1 is sufficient to drive the mammalian cell cycle. Nature.

[53] Lee MG, Nurse P (1987). Complementation used to clone a human homologue of the fission yeast cell cycle control gene cdc2. Nature.

[54] Mendenhall MD, Hodge AE (1998). Regulation of Cdc28 cyclin-dependent protein kinase activity during the cell cycle of the yeast *Saccharomyces cerevisiae*. Microbiol. Mol. Biol. Rev..

[55] Huang D, Friesen H, Andrews B (2007). Pho85, a multifunctional cyclin-dependent protein kinase in budding yeast. Mol. Microbiol..

[56] Carroll AS, O’Shea EK (2002). Pho85 and signaling environmental conditions. Trends Biochem. Sci..

[57] Li S (1995). The cdc2-related kinase, PISSLRE, is essential for cell growth and acts in G_2_ phase of the cell cycle. Cancer Res..

[58] Malumbres M (2014). Cyclin-dependent kinases. Genome Biol..

[59] Ma Z (2013). Phylogenetic analysis reveals the evolution and diversification of cyclins in eukaryotes. Mol. Phylogenet. Evol..

[60] Schachter MM (2013). A CDK7-CDK4 T-loop phosphorylation cascade promotes G_1_ progression. Mol. Cell.

[61] Larochelle S (2007). Requirements for CDK7 in the assembly of CDK1/cyclin B and activation of CDK2 revealed by chemical genetics in human cells. Mol. Cell.

[62] Jones NG (2014). Regulators of *Trypanosoma brucei* cell cycle progression and differentiation identified using a kinome-wide RNAi screen. PLoS Pathog..

[63] Tu X, Wang CC (2005). Pairwise knockdowns of cdc2-related kinases (CRKs) in *Trypanosoma brucei* identified the CRKs for G_1_/S and G_2_/M transitions and demonstrated distinctive cytokinetic regulations between two developmental stages of the organism. Eukaryot. Cell.

[64] Ortega S, Malumbres M, Barbacid M (2002). Cyclin D-dependent kinases, INK4 inhibitors and cancer. Biochim. Biophys. Acta.

[65] Roques M (2015). *Plasmodium* P-type cyclin CYC3 modulates endomitotic growth during oocyst development in mosquitoes. PLoS Pathog..

[66] Peng L, Skylar A, Chang PL, Bisova K, Wu X (2014). CYCP2; 1 integrates genetic and nutritional information to promote meristem cell division in *Arabidopsis*. Dev. Biol..

[67] Huang D (1998). Cyclin partners determine Pho85 protein kinase substrate specificity *in vitro* and *in vivo*: control of glycogen biosynthesis by Pcl8 and Pcl10. Mol. Cell. Biol..

[68] Barott KL, Venn AA, Perez SO, Tambutté S, Tresguerres M (2015). Coral host cells acidify symbiotic algal microenvironment to promote photosynthesis. Proc. Natl. Acad. Sci..

[69] Alvarez CA, Suvorova ES (2017). Checkpoints of apicomplexan cell division identified in *Toxoplasma gondii*. PLoS Pathog..

[70] Measday V (1997). A family of cyclin-like proteins that interact with the Pho85 cyclin-dependent kinase. Mol. Cell. Biol..

[71] Jiang M, Gao Y, Yang T, Zhu X, Chen J (2009). Cyclin Y, a novel membrane-associated cyclin, interacts with PFTK1. FEBS Lett..

[72] Sun T, Co NN, Wong N (2014). PFTK1 interacts with cyclin Y to activate non-canonical Wnt signaling in hepatocellular carcinoma. Biochem. Biophys. Res. Commun..

[73] MacDonald BT, Tamai K, He X (2009). Wnt/β-catenin signaling: components, mechanisms, and diseases. Dev. Cell.

[74] Leander BS, Keeling PJ (2004). Early evolutionary history of Dinoflagellates and Apicomplexans (Alveolata) as inferred from Hsp90 and actin phylogenies. J. Phycol..

[75] Liu Y, Hu H, Li Z (2013). The cooperative roles of Pho80-like cyclins in regulating the G_1_/S transition and posterior cytoskeletal morphogenesis in *Trypanosoma brucei*. Mol. Microbiol..

[76] Stat M, Gates RD (2010). Clade D *Symbiodinium* in scleractinian corals: a “nugget” of hope, a selfish opportunist, an ominous sign, or all of the above?. J. Mar. Biol..

[77] Keeling PJ (2014). The Marine Microbial Eukaryote Transcriptome Sequencing Project (MMETSP): illuminating the functional diversity of eukaryotic life in the oceans through transcriptome sequencing. PLoS Biol..

[78] Duncan RP, Feng H, Nguyen DM, Wilson ACC (2016). Gene family expansions in aphids maintained by endosymbiotic and nonsymbiotic traits. Genome Biol. Evol..

[79] Chen Y, González-Pech RA, Stephens TG, Bhattacharya D, Chan CX (2019). Evidence that inconsistent gene prediction can mislead analysis of dinoflagellate genomes. J. Phycol..

