# Supplementary material for: Phylogenetic analysis of cell-cycle regulatory proteins within the Symbiodiniaceae
Source: Sci Rep. 2020 Nov 24;10:20473. doi: 10.1038/s41598-020-76621-1 (PMC7686383; doi:10.1038/s41598-020-76621-1)
Supplement: Supplementary file 1 — Supplementary Information 1. [file 41598_2020_76621_MOESM1_ESM.docx]

**Phylogenetic analysis of cell-cycle regulatory proteins within the Symbiodiniaceae**

Lucy M. Gorman^1^, Shaun P. Wilkinson^1^, Sheila A. Kitchen^2^, Clinton A. Oakley^1^, Arthur R. Grossman^3^, Virginia M. Weis^4^, Simon K. Davy^1*^

^1^School of Biological Sciences, Victoria University of Wellington, Wellington 6140, New Zealand

^2^ Division of Biology and Biological Engineering, California Institute of Technology, Pasadena, California 91125, United States

^3^Department of Plant Biology, The Carnegie Institution for Science, Stanford, California 94305, United States

^4^Department of Integrative Biology, Oregon State University, Corvallis, Oregon 97331, United States

*Corresponding author: Simon.Davy@vuw.ac.nz
