# Supplementary material for: Phylogenetic analysis of cell-cycle regulatory proteins within the Symbiodiniaceae
Source: Sci Rep. 2020 Nov 24;10:20473. doi: 10.1038/s41598-020-76621-1 (PMC7686383; doi:10.1038/s41598-020-76621-1)
Supplement: Supplementary file 4 — Supplementary Information 2. [file 41598_2020_76621_MOESM4_ESM.docx]

**Phylogenetic analysis of cell-cycle regulatory proteins within the Symbiodiniaceae**

Lucy M. Gorman^1^, Shaun P. Wilkinson^1^, Sheila A. Kitchen^2^, Clinton A. Oakley^1^, Arthur R. Grossman^3^, Virginia M. Weis^4^, Simon K. Davy^1*^

^1^School of Biological Sciences, Victoria University of Wellington, Wellington 6140, New Zealand

^2^ Division of Biology and Biological Engineering, California Institute of Technology, Pasadena, California 91125, United States

^3^Department of Plant Biology, The Carnegie Institution for Science, Stanford, California 94305, United States

^4^Department of Integrative Biology, Oregon State University, Corvallis, Oregon 97331, United States

*Corresponding author: Simon.Davy@vuw.ac.nz

**Figure S1. A.** Residues 1-10 of trained cyclin pHMM model for querying Symbiodiniaceae databases. **B.** Residues 1-10 of trained cyclin-dependent kinase pHMM model for querying Symbiodiniaceae databases. Black horizontal bars represent the likelihood of an amino acid appearing at that residue. Match states are shown as rectangles, insert states as diamonds, and delete states as circles. Numbers in the delete states are simply model module numbers, while those in the insert states are the probabilities of remaining in the current insert state at the next emission cycle. Lines are weighted and directed where necessary to reflect the transition probabilities between states. The large “B” label is the silent begin state of the model.

**Figure S2.** Full phylogenetic tree of CDKs within Symbiodiniaceae. Colour of branches corresponds to aLRT support (SH-value). Purple branches correspond to SH-values below 0.5, brown branches correspond to SH-values near 0.5, and green branches correspond to SH-values close to 1. Symbiodiniaceae species are written in blue. The tree was made using PhyML(v3.1)^S1^ and visualised using the Interactive Tree of Life software (v.5.6.3)^S2^.

**Figure S3.** Full phylogenetic tree of cyclins within Symbiodiniaceae. Colour of branches corresponds to aLRT support (SH-value). Purple branches correspond to SH-values below 0.5, brown branches correspond to SH-values near 0.5, and green branches correspond to SH-values close to 1. Symbiodiniaceae species are written in blue. The tree was made using PhyML(v3.1) ^S1^ and visualised using the Interactive Tree of Life software (v.5.6.3)^S2^.

**Table S1.** Cyclin- and CDK-specific roles and timing within the mammalian cell cycle. Class I refers to those involved directly in the cell cycle and Class II refers to those involved in transcription.

|  | Class (I/II) | Cell-cycle stage | Role |
| --- | --- | --- | --- |
| CDK1 -Cyclin A/B | I | S/ G_2_/M | CDK1 sequentially binds to cyclin A in the S phase and then binds to cyclin B in the M phase. Binding to cyclin B triggers the mitotic cascade ^S3^. |
| CDK1 - Cyclin J | I | N/A | Expressed within ovaries and functions in egg chamber development in *Drosophila* ^S4^ |
| CDK2 - Cyclin A | I | S | Phosphorylates replication substrates in S phase ^S5,6^. At the end of interphase, cyclin A activates CDK1 which is essential for progression into mitosis ^S7^. |
| CDK2 - Cyclin E | I | S | Phosphorylates retinoblastoma (Rb) and promotes S phase entry ^S8,9^ |
| CDK3 | I | G_0_/G_1_ | Phosphorylates retinoblastoma protein (pRb) leading to the expression of cyclins and CDKs, allowing quiescent cells to re-enter the cell cycle ^S10^ |
| CDK4/6 - Cyclin D | I | G_1_ | Mitogenic sensors that pick up on extracellular cues (e.g. growth factors and nutrients) to elicit cell cycle progression ^S11–14^ |
| CDK5 | I | N/A | Activated by p25/p35 and p29/p39 and targets retinoblastoma protein (pRb) downstream ^S15^ |
| CDK5 - Cyclin D/E | I | N/A | Cyclin D and E binding to CDK5 reduces its activity ^S15^ |
| CDK5 - cyclin I | I |  | Abundant in post-mitotic cells and serves anti-apoptotic/pro-survival function ^S16–18^ |
| CDK7 - Cyclin H | II | N/A | Forms the CDK-activating kinase (CAK) that activates the cell-cycle CDKs1/2/4/6 ^S10^ |
| CDK8 -Cyclin C | II | N/A | Initiates gene transcription by phosphorylating heptad repeats in RNA polymerase II ^S10^ |
| CDK9 -Cyclin T/Cyclin K | II | N/A | Catalytic subunit of positive transcription elongation factor b (P-TEFb) which targets the carboxyl-terminal domain (CTD) of RNA polymerase II and is essential for the synthesis of mature mRNA ^S19^ |
| CDK10 - Cyclin M | II | G_2_/M | Forms a complex with CDK10 ^S20^ and regulates gene transcription by phosphorylating diverse substrates ^S10^ |
| CDK11 - Cyclin L | II |  | Forms a complex with CDK11 and regulates RNA splicing ^S10^ |
| CDK12 - Cyclin K | II | N/A | Initiates gene transcription by phosphorylating heptad repeats in RNA polymerase II ^10^. Regulates genes involved in the DNA damage response ^21^ |
| CDK 13 - Cyclin K | II |  | Initiates gene transcription by phosphorylating heptad repeats in RNA polymerase II ^10^ |
| Cyclin F | I | S/G_2_ | Restricts E2F activity to the S phase which: prevents premature S phase entry; inhibits DNA damage; and, increases cell fitness ^22^ |
| Cyclin G | I | G_2_/M | Triggers cell cycle arrest in response to DNA damage ^23^ |

T**able S2.** Symbiodiniaceae genome and transcriptome databases used.

| **Database** | **ITS2 type** | **Transcriptome (T) Genome (G)** | **Assembly** | **Gene number** | **Contig N50 (bp)** | **Source** | **Reference** |
| --- | --- | --- | --- | --- | --- | --- | --- |
| *Symbiodinium tridacnidorum* (CCMP2430) | A3 | T | MMETSP1117 | 39,104 | 1,163 | iMicrobe - Marine Microbial Eukaryote Transcriptome Sequencing | Keeling *et al.* 2014 ^S24^ |
|  |  |  | MMETSP1115 | 47,757 | 1,551 |  |  |
|  |  |  | MMETSP1116 | 40,140 | 1,187 |  |  |
| *Cladocopium* sp. #2 | C | T | MMETSP1122 | 47,710 | 1,551 |  |  |
|  |  |  | MMETSP1123 | 37,758 | 1,129 |  |  |
|  |  |  | MMETSP1124 | 35,273 | 1,123 |  |  |
|  |  |  | MMETSP1125 | 40,531 | 1,355 |  |  |
| *Cladocopium* *goreaui* | C1 | T | MMETSP1367 | 48,210 | 1,424 |  |  |
|  |  |  | MMETSP1369 | 48,216 | 1,377 |  |  |
| *Cladocopium* sp. C15 | C15 | T | MMETSP1370 | 44,616 | 1,194 |  |  |
|  |  |  | MMETSP1371 | 51,910 | 1,190 |  |  |
| *Symbiodinium* sp. #2 | A | T | MMETSP1374 | 43,062 | 1,297 |  |  |
| *Durusdinium trenchii* | D1a | T | MMETSP1377 | 56,916 | 852 |  |  |
| *Fugacium kawagutii* (CCMP2468) | F | T | MMETSP0132_2C | 18,489 | 214 |  |  |
| *Effrenium voratum* (CCMP421) | E | T | MMETSP1110 | 77,821 | 1,725 |  |  |
| *C. goreaui* | C1 | G |  | 35,913 | 6,576 | Reef genomics | Liu *et al.* 2018^S25^ |
| *F. kawagutii* | F | G |  | 26,609 | 35,743 |  |  |
| *B. aenigmaticum* | B1 | T |  | 45,343 | 1,355 | Reef genomics | Parkinson *et al.* 2016 ^S26^ |
| *B. pseudominutum* | B1 | T |  | 47,411 | 1,508 | Reef genomics |  |
| *B. psygmophilum* | B2 | T |  | 50,745 | 1,618 | Reef genomics |  |
| *B. minutum* | B1 | T |  | 51,199 | 1,579 | Reef genomics |  |
| *B. minutum* | B1 | G |  | 47,014 | 2,675 | Shoguchi *et al.* 2013 ^S27^ | |
| *S. microadriaticum* | A | G |  | 49,109 | 3,987 | Reef genomics | Aranda *et al.* 2016 ^S28^ |
| *Symbiodinium* sp. #1 (CassKB8) | A | T |  | 57,676 | 1,087 | Bayer *et al.* 2012 ^S29^ | |
| *B. minutum* | B1 | T |  | 56,198 | 741 |  |  |
| *Cladocopium* sp. #3 | C | T |  | 26,986 | 534 | Ladner *et al.* 2012 ^S30^ | |
| *Durusdinium* sp. #1 | D | T |  | 23,777 | 920 |  |  |
| *Cladocopium* sp. #1 | C | T |  | 55,588 | 687 | González-Pech *et al.* 2017 ^S31^ | |
| *Cladocopium* *goreaui* – MI population | C1 | T |  | 106,097 | 1,239 | Levin *et al.* 2016 ^S32^ | |
| *Cladocopium* *goreaui* – SM population | C1 | T |  | 93,377 | 1,323 |  |  |
| *Breviolum* sp. #1 (SSB01) | B | T |  | 59,669 | 1,752 | Xiang *et al.* 2015 ^S33^ | |
| *Fugacium kawagutii* | F | G |  | 36,850 | 1,467 | Lin *et al.* 2015 ^S34^ | |
| *Symbiodinium tridacnidorum* | A3 | G |  | 69,018 | 1,774 | Shoguchi *et al.* 2018 ^S35^ | |
| *Cladocopium* sp. C92 | C92 | G |  | 65,832 | 1,686 |  |  |

**Table S3.** CDK motifs used to scan Symbiodiniaceae databases.

| CDK Motif |
| --- |
| PSTAIRE |
| PTTAIRE |
| PATAIRE |
| PTSFLRE |
| PSTALRE |
| PATALRE |
| PVSSIRE |
| PAVAMRE |
| PAVALRE |
| PRISLRE |
| PVSLLRE |
| PAHVLRE |
| PEQFQEE |
| PEKLKEE |
| PLSLLRE |
| PPYALRE |
| PSSSLRE |
| PEILERE |
| PLSSQRE |
| PQEVQRE |
| PSEIANE |
| PARFQRE |
| PARFQVE |
| PLCVERE |
| PSASLRE |
| PAVIRRE |
| PAATIRE |
| PCTAIRE |
| PISTVRE |
| PSSALRE |
| PLSTIRE |
| NRTALRE |
| SMSACRE |
| PITALRE |
| PISSLRE |
| PITSLRE |
| PFTAIRE |
| PNQALRE |
| PPTALRE |
| PITAIRE |
| NFTALRE |
| SPTAIRE |
| SCTTLRE |
| PLTNLRE |
| PKNAIRE |
| HFTTLRE |
| PTSSLRE |

**Table S4**. Candidate proteins used for custom BLAST searches performed on Symbiodiniaceae databases

| Protein relation | Species | Database | Candidate gene ID | Candidate gene |
| --- | --- | --- | --- | --- |
| Cyclin A | *Cladocopium goreaui* | MMETSP1367/1369^S24^ | CAMPEP_0199578094 | XMCATEVALSLKWPDNSHLMAPAGAACLPPKALAPRAAYHSRSGSLPALEVESTSGAIEHSGLDTRGAPDQGPKSCKGLKSMSVPKGRPAASTVLSLPVGGGTKMAAKVSSKALVGSAMAESAVPEPSTSMPVEDWTDVDKLNEMDPLAVSEYAQSICQHLRESELVKRPSSSYLERVQGDVNAKMRAILVDWLVEVTEEYTLCADTLYQAVNYIDRFLSTRVTTRAELQLVGVTCMWLSSKYEEIYPPTVSDFCFITDNTYTREQLIEMEEVVLKELKYELTVPTAKTFLRRMLQVCSPDELLHFLSNYLTELSLLDYAMLRYLPSTIAAAAIYLANVMLGREPWSANLRHYSTYAPEDIEECVLALAAVHKAATACPSLAAIRDKYAHPRFHEVSMISPVTAAAVTATL |
| Cyclin B | *Durusdinium trenchii* | MMETSP1377^S24^ | CAMPEP_0196951544 | HVNDPALVASYSADIYQYMREREECLLIDPNFLQRQSRVTAKNLAVLHDWLVQVHYKFELQLETLYITNAILLRYLSRVDTPRSKLQLYGVTAMLLASKYEDMYPPVVRDFAYITANAYKPREIIKAEMEMLTTLEFSLEQPLPLX |
| Cyclin D | *Cladocopium* sp. C15 | MMETSP1370/1371^S24^ | CAMPEP_0192418040 | MELLCCEGPRVRYAYQDPVLLQDERVLRNLLTCEDKYIPSCRYFNIVQKEIEPHMRRMVTSWMLEVCEEQMCEEEVFPLAVNYLDRFLSVVPTRKCQLQLLGAVCMFIASKLKETSPLPAEKLCIYTDNSITCQELLDWEILVLGKLKWDLSAVTPYDFLEQIFSRLSLPNVSVIRKHAATFIALCCTDEKFLMYPPSMLAAASVCAAFTGLATEEQKSVWTRPMLFSFLQGLTNIEPEYLQSCQELMEEVLHFNVTEPPTSKVENGCSPSTPTDLQEIHF |
| Cyclin G/I | *Cladocopium* sp. C15 | MMETSP1370/1371^S24^ | CAMPEP_0192409796 | MKVSCGLNVGKLLRVLQEGLLKEEAAHFAPLTCLVGNEDSDGISLSQRDNITTFMLNLSRRCGFHSETYSLSVNLLDRFLSVVKANPKYLPCMSICCLFLAIKMSEEDEDVPTAADFVKVSGLRFSSSDLLRMERIILDKLNWNLNATTPLYFLQVFHALGVAKGFLDHCPVNQHLQHITSLMEGLLCHHKFMFFKPSTLALALLSHELVYVSNNWFMATHYLQHEGKVSDAELWACSKLVNEHLNSVIQKHLPSFKTLPVTENEDKEFPVIEN |
| Cyclin F | *Symbiodinium* sp. #2 | MMETSP1374^S24^ | CAMPEP_0196858170 | MVIESAPPSMFRQFVTRNTRPRRDPLDIRRGHHEAMMRSRSANPRAPLGDITNGVQADLPVKRRSLAPGDPASTALLDGPVWPRVEYSTDFMLNMLDAERKHRQPSADYMRHQNVVHEGMRSVLIDWLVDVHATYELRIETLFLTISIIDQYLAQVKVSRRELQLIGVASMFIASKFEEIHPPEAKDFVYITAKSYSKQEIFDMELRILSQLQFKVARPTVAHFLQRLEAEASRASKPDSPRERVMQHLPWYLVELCMLDVGTLQFMPSCVAVAALTLTRRLLNVNAAGPHQCMDLVGQTLAVEVLEECMNFMLNLLEAAPSTATTAAVRRKHSDQISVLGAPQEETEQAVAI |
| Cyclin L | *Cladocopium goreaui* | SM population – Levin *et al.* 2016 ^S32^ | C1SM_TR46552_c0_g1_i1_m.62470 | YSSPQPSGGPGSALMLALVPEDVLAAPPSREDGIDEDSEDQLRRFGANLIQRAGVLLRLPQLSVATASGLFQRFYFRKSFAEFEVRALAMASLTLASKLLEHPRKVVDVIQVFYKLKMREAQEQDGSASFAGMPTPLLDPTKKEFHDAKKELLSAERNILRELGFEVHLLLDHPHRYAIEYIEHLQRPAELTQKVWNYLNDALQTSLCCAHQPRNIAGASLVLASKELGVNLPSKPPWWETFGVQIKDAELIANEMEELYQKKRPEYIEIPRRKREVFEPMTPFPSPPSGPGKSPSEEDDHVDGETSLARQDSNIDLEGLEEAMAQSVAALARAKEGSPQRERPGEDKKEKLVENKLAQEQPQNAQHERSAKDRKKRDRQSEGSQSPKRKNAKKTRGS* |
| Cyclin P/U | *Breviolum minutum* | Parkinson *et al.* 2016 ^S26^ | >m.6019 g.6019 ORF g.6019 m.6019 type:5prime_partial len:218 (-) comp9819_c0_seq1:142-795(-) | KTHLIFLQRRTWVAGHARACRPTGTVGVSMEMEDEQEDEPAYDDPGQQVANAGQSFVLALADVLTHLSSLRPPPTGQRVTKFHSVRPPQLPIRDYLFRIARYFQCSRECFVLCLVYIDRIVKLHPDFTICSLNIHRLLVTSVMLAVKFFDDVYYSNAYYAKVGGVRTKEVNALESHFLQLIEWKLHVTPEEFDQYRSHVCTVGAAQPVPRLADDALG* |
| Apicomplexan Mitotic Cyclin | *Cladocopium goreaui* | MI population – Levin *et al.* 2016 ^S32^ | C1MI_TR14393_c0_g1_i1_m.19980 | FRTRDDNAVNKPPGSTAVTGDVVTRAPVMTQPPISANSRDALPHHVVEYADEIVQHLLEREQILFAMRAPDYLSAQPDVTERMRIILVDWLVDVHLKFKLHPETFFLAVDYVDRYLMTTKGERSTLQLIGVTAMLIAAKHEEIWPPEVKECVYISANTYQHQEILNMERDIVSALNFKLCVPTPYPFMLRLVEGTDATQDTRHLASYCLDLSSLDYSCLKFLPSTVGFASVLIANLVAENTRRSSNGRHPVAVPLISASDDAEALWTDEHGALSSIDRSQLGIVIECARSILGCASNVNTPTSRYHAVRRKYSSERFGEVASRYTLPPTI* |
| Dinoflagellate-specific Cyclin #1 | *Cladocopium goreaui* | MMETSP1367/1369^S24^ | CAMPEP_0199568816 | MAFQARRHANSENVNPNVGATRLGAPQARRKVATAKPQRGVNRAPLASLADITNIQDARDMRKKPLREPLAPLAPLAPLERSLVTIRNREASPTPMEISNPELLAEAHDKVQSVAEYAPEIADQLFHDEAIFMPRADYMESQQDINGKMRAILVDWLVEVHMKYRLRPETLFLAVNLIDRYMSSLPVLRRRLQLLGVVAMFVAAKFEEIDPPKATDFVYITDNTYSKDELFQMECNMLSTLEFQVVVPTAAHFVNQFVKANGCENPRHAEVIKYIMELALLDLRMIRHKASHLVAAAVLLSNELFGRAIPWPEHMIQISRHTDAELRVCCEELRQLVRQAPSQQLQAVRKKYMLAQHYQVARNAVLVAAXSHPMSFDFGRCWRRVAGARCNIGAASPRWRRVLANVGX |
| Dinoflagellate-specific Cyclin #2 | *Cladocopium goreaui* | MI population – Levin *et al.* 2016 ^S32^ | C1MI_TR28979_c0_g1_i1_m.38873 | ADLGKATAGGGHWSISMTRATSDHSVRSAAHLNILPVAMQPQVRPARRNAVLGDITNTGPIGLGASGKVLGSEKSSLPSFAPMPSLKTTSVMGTGTTSAAGRCFRGKEDGQLGLFSLGSAPQAHAREQHARFIRPASGDFMDVAMEEEPSEDPQHVAEYTKDIYAHMFAIEGSFQPRPHYLTEQREINAKMRAILVDWLVEVHMKHRLRRETLFMAVSLIDRYLSVRQVARKRLQLCGVAAMFIAAKFEEIYPPEVKDFVYITDNAYTKDDILNMEVSMLRTLDFALCGPTAAHFLDRFHRANVCSEEQLHLMHYLAELALLEVQMLQYTPSHIAAAAALLSNKLLKFPTWPPCMAQFSKHSEGEIKACARELCGILESVDRSSLQAIRKKYSQERFKRVAKLSFGSS* |
| Dinoflagellate-specific Cyclin #3 | *Cladocopium goreaui* | MMETSP1367/1369^S24^ | CAMPEP_0199563964 | MKRVLADITNELRLSPVRKVPREVHLEEFSDFTSPDVRGLKGWDDQELLHRRLARLQLSPEPCGIDDDDPQGVAEYVADIFSKLEDDEIYHLSPQGVQLGRLWERERATAVDWMVEVQVLYGLRTETLFLAVSLLDSFLKLNEVNQVQLQLAVVCSLFVAAKFEEIEPPNVKDFVNMTNEVCNKQDILAMEATLLTSLEFSLCRPTAVHFLERGSRSPQRLFSRKMLQKHGFLTQYLLELALVDSQMLRFPPSLQVAAATMVSSRLLGSLVRVPRHDISGERSAMIYRCALEMCRLLEEVELSSHQAVRKKFLRPDYLSVAAMVSCT |
| Unidentified cyclin #1 | *Symbiodinium* sp. #2 | MMETSP1374^S24^ | CAMPEP_0196853782 | XSRPPLRFFMEHRHLDAEAHKGMLRMIRSARSRLNKEAEDPSANFTSAVADGRDGGLPVSMHLSSRWRLEAGSLHQELKTARTDRSESVGCTRPHASLLQHAVATGDSPTHPVQRMRSDQRAVALEPQQPPLNDRALKRGGQRSTFGGVGRPRLARSRSTADALLATQGASENAVPAIQQADAPALPASQPLPHPAPQFVLSDVTNTAGHTGPPAAKLHAQTEPDANCVKPSVPPPTVTEPCAGHPAVASAVPSAPPLAPRGAPAPSRTAGGPRPSVVVAAQSPESRPSTEATDMEVDAVGIDAEDPQVPVEYLADIYRHLDREEAHRLPRVLYMEKQTHVNAKMRAILIDWLVDVHKKYKLQTETLFLATSVVDGFLEQRVVQRRHLQLVGVTGLLIAAKFEEMYPPQINDFVYVTAKAYKKEEVARMEVSMLNALDFNLCHPTAAHFLARYQCVNGCSEAHADLAQYLLELTLVDYKMIRYAPSHLAAAAILLSNKLLRRQPCWPASVVRHTKLTESALKDCAKEICAALEQAENNPLQAVRKKFSQQKYHSVAKLNFTAAPSYVHAREGARRTSVRRSTANGSSQDSPSGQARQPSEGNPV |
| Unidentified cyclin #2 | *Cladocopium goreaui* | MI population – Levin *et al.* 2016 ^S32^ | C1MI_TR17217_c0_g1_i1_m.23333 | MMDREDMMIEENNLSLEDIDEYDREDPQFCTEYVEEIFALLREKEQTNRVEAGYMANQDDLIPSYRTKIVNWMGEVYMKFRLLSETLILAVNILDRFLMERPVSRSRLQLLGATAMLVACKFEEIYLPQIDDFVYLCADAYSRKDFLRMENIILGTLNYNLAVPTPLHFLRRFSKAAFSDRKVHTLSKYITELSLSSYELLRFLPSQVAAAAVLVARNMSGITPLWNSTLRHYTQYKESDIMECAEMLNEMIRQVHEEA |
|  | | | | |
| CDK1/2/3 | *Cladocopium goreaui* | SM population – Levin *et al.* 2016 ^S32^ | >C1SM_TR64037_c0_g1_i1_m.108940 | EKRIEKGKMSIMEKYVKVEKPVGEGTYGVVYKARHKETGDIVALKKIRLEMEDEGVPSTALREISLLKELDHPNIVRLRDVEHQQQPKRLYLVFEWLEQDLRKHMDNLDGPMSNELIKSYMSQMLQGLDYCHCHGIFHRDLKPQNLLIDRTGTLKIADFGLARAFSLPFRTYTHEVVTLWYRAPEILLGQRRYGLPVDMWSVGTILAEMSNRRPLWPGECEIDELYKIFRSLGTPDDSMWPGVASLPDYQDVFPNWSPQPLEKDVPRLEPMGIKLLAEMLKYDPATRISARNALRHEYFKDM* |
| CDK5 | *Durusdinium trenchii* | MMETSP1377 ^S24^ | CAMPEP_0196914612 | LLYKTYLLFFFLVVFISMISKSKLDKYEKLDKLGEGTYGVVYKAKDKTTGDLFALKKIRLESEDEGIPSTAIREIALLKELQHPNIVRIHDVIHTNKKLILVFEYVDYDLKKFLNSFDKGIDIKIAKSLLYQLVRGIAHCHQMRVLHRDLKPQNLLVSKEGVLKLADFGLARAFGIPVKNYTNEVVTLWYRAPDILLGSKNYSTTVDIWSIGCIFVEMLNLKPLFPGSSEPDQLKKIFKIMGTPDPEKWPGLTELPDYKPENFEGYTTEPLNKLCPSMPEDGLDLLDKMLRCNPAERITAKDALKHKFFEDIPENLKKLYN |
| CDK10 | *Cladocopium goreaui* | Liu *et al.* 2018^S25^ | SymbC1.scaffold196.6 SymbC1.scaffold196:164432-177259(+) | MSGLESGTYGTVYRARDTETGDIVALKKVRIHAEKEGFPRISLREIRLLKRLRHPNIVELREVACGRQSGSVFLVFEYCEHDVGALLDLMERPFSQPEVKCLTLQLLKAVECLHLASVIHRDIKLSNLLLNNKGVLKLADFGLAREFVDFQTPITQNVVTLWYRAPELLFGAKKYTVAVDMWSVGCNFGELLLKRPLLPGKCEEHQLVLTCELLGTPTPRIWPGVEKLPHYAASKLPENIYNNLGLKFPDLPDSCLDLLNRLLTFDPQKRSSASSSLQHLWFSEAPAPQEPHYMPTFREHRNETANPRGLPAAAKAPAKRPMVARSAVFAAAKKLKSCVF |
| CDK11 | *Cladocopium goreaui* | Liu *et al.* 2018^S25^ | SymbC1.scaffold1236.5 SymbC1.scaffold1236:119372-126370(-) | MATLDAADGEAAAKRQRVGGWAENCLNQGCRSVQCFRKLNRIDEGTYGVVYRACEIDTGEVVALKQLKLGAVKSEEGFPVSSIREISLLLELNHPNVVQCREVVLGNTMQHVYMVMEYVEHELKVLITQQRFAVAEMKCLLRQLLLGLAHLHAMWIVHRDLKTSNILLDRNGILKICDFGLARHFGQPLRPYTHRVQSLWYRAPELLLGQRTYSNAIDVWSSGCIFAEMLLRRPVFEGKAEMHQLGLIMGLVGLPDEESWPGCSELPHWKMLESFKDTMPGWRELFPEPPDSTLSELGLLLMRGLLECCPARRLAAADAVEHHYFQEVPQPQEPSMLPTFKESNSSTRGQR |
| CDK12/13 | *Breviolum minutum* | Shogucchi *et al.* 2013 ^27^ | symbB.v1.2.023129.t1 | MSTGKYQKVDDSPVGEGTYGTVWKGVNRENSAEVAMKKVVIRHPKEGLPTTAIREIRALRTLQSHPNVVKMYDVYSEMPGSNGSVGDVYLIFEYAPHDLTGFMAYRKKLKLTEIKCLTAQLLEGLDYCHSLLVMHRDLKPSNILLTADGTLKLCDFGLCRLVKEAEPGAYTTRVITLWYRPPELLLGCQKYDFSVDIWSAGCIVGEMLFTVPLFPDSAEVQVLKKIRNRLTAFNADDWPSSMRKHQHWEKFWQQINRPVAPGENRDLYGDLKVKHGSLCVDFLKSFIHLDPAERKDTGTLLNHEFLDEEPLACGKKEMKMPPEGTNMKELGIKRKAEEAGHGGKQRAPKRHADEGRLEPSPKRPRAP |
| Alveolate-specific CDKA | *Cladocopium goreaui* | Liu *et al.* 2017^S25^ | SymbC1.scaffold591.6 SymbC1.scaffold591:142736-143969(+) | MQVLADAGRSLLTERHLPELPDSPATDDRPDGADCSDVLLQAALHLQPWAVRGMEQYQKIEKVGEGTYGVVYKAQDSGGKVYALKTIRLEAEDEGIPSTAIREISLLKELQHPNIVRLCDVIHTERKLTLVFEYLDQDLKKLLDMCEGGLDSATTKSFLYQLLRGIAYCHAHRVLHRDLKPQNLLINREGSLKLADFGLARAFGIPVRSYTHEVVTLWYRAPDVLMGSRKYSTPVDIWSVGCIFAEMVNGRPLFPGDTDANQLQKIFRILGTPSAETWPTITELPDWKPDFPVFEPQAWTSITPTLEPEGMDLMTKFLQYWPDRRISGKAAQEHDYFKELSDAIKNMK |
| Alveolate-specific CDKB | *Cladocopium goreaui* | Liu *et al.* 2017^S25^ | >SymbC1.scaffold10778.1 SymbC1.scaffold10778:323-3022(+) | MALKPRSSLEDFHGVENISHNLGDDEENMQPGRQTPISQRRPLQQRFNFNCMALSPTKCRKQSHGLAEESPLKLAKDEVWISPAKLPEMRSGTFSLWLEVFRRLGARDIVSKAAPVCRQWRDVAQDRELWALARQHLRLVDCHVMLDKVVERRSKGRIFKCRALGSGDIVMLRMVDLELTNAGRDDGMPTSFLREAALLSELRHPNVIRHYGAEILDKRGVVCSEFVYENWTSWFKRLEVKFPCQRMEDIKGNFSQMLRGLNYLHHQGLMHRNLKPDNIFIDELGTVKVGDFTTTRMLDIPFQAYTPEDPKERDRSGREMRRLWYRSPELIIREEIYGPKVDTWSVGCLFVEAATGRPLFQSDSEIDHLFRIFRLVGTPTLANWPGVVAAKNFSPKFPMYQGFSFAQVARAESLKPQSFEDQQRLWLQAQPDREEMLHQLIQIARVVGVDGMFLLDRLITAAPLSRAGVEETLRMPFFAPSFGSEQGSLGSFGQGAQGRLQRNVQSFHPMTELWLGGRPVRLEEQREQRPQPKNLENPEAKSYATPPTPATTLANIGSAQSQYPPMAIPSSLITSEMVWNILNVMLEQERSPSSVFATWSLPPGFDANARAVQVDFIIGLASSMNLRASTAHLACAVFDKYLSLQEKPVMPEQIKVVAATCLKVSDIFGEQSKEYYKQENSVEYTEAAVGKSITPSQMLSCEKEILPKLGFKLHHPTIRWFLQCYIAYARLSMFDAVGKTASFIADLMLLDFELLLYTPSLKAQCAVLMAAFLVQQEATLHQPHQPLDKAMKSLPNEDQSDKCKSIGPLQGYLSCLAYWDKNIRDAVCRANVAVDASMCLQAVVRMLLDKRREWKSLQLNAVEIKHAQLARALAYPDRFPVFKLLRYILSDHQRSLVPE |
| Dinoflagellate-specific CDKA | *Breviolum aenigmaticum* | Parkinson *et al.* 2016 ^S26^ | >m.43804 g.43804 ORF g.43804 m.43804 type:5prime_partial len:303 (-) comp28229_c0_seq1:90-998(-) | KLGEGTYGKVYKAACHQTGQVVALKRIPIVMDEDGVPATAIREVSLLKECDHPNVIRLHEVLSLDRALYLVFEYVDMDLRIFLKRNGAFKDPLALKNAAWQCIRGTAFCHGRQVLHRDLKPQNVLVDSTGCHLKLADFGLARLLDVPLRAYTHEVVTLWYRAPEILLGHRKYAMPTDIWSLGCIVAEMATAEVLFPGDSQIDTIFKIFRRLGTPSEEVWPGFSTLKNFTEEFPKWSNTELVDVRSKAPSLGSRGVDMINACLRFNPVDRPSALKLLQHKFFERAPLYEAVAVAEEAGRTSSC* |
| Dinoflagellate-specific CDKB | *Breviolum minutum* | Parkinson *et al.* 2016 ^S26^ | >m.48530 g.48530 ORF g.48530 m.48530 type:5prime_partial len:339 (-) comp39104_c0_seq1:385-1401(-) | LTIQRNVSFDMLGGRSHPMQRSMADLEDNLDEAEKAFESQYEKVEPSLLGEGTYGKVFKAKSIRTGELVAMKQMKLEGSEDGMPSTALREIALLKELKDHQNIVRLLNIFYKPNKLVLVFEFVENDLKKYMRSMGNNLSPGTVKNFAFQLFQGVQFCHANRILHRDLKPQNLLIDQRLRLKIADFGLARPFHVPVGEYTHEVVTVWYRPPEILLGSQKYSLPVDLWSIGCVIAEMATGSALFPGDSEIATIFKIFQRLGTPTEQMWPDITKLPYFKPSFPQWPAHSWSQIRNTLQQVGSDGCDLLDKLTYYDPRRRISAHRALQHAYFRDIDPRDGEV* |
| Dinoflagellate-specific CDKC | *Breviolum pseudominutum* | Parkinson *et al.* 2016 ^S26^ | >pmin_comp22611_c0_seq1:82-1323(-) | MGFMETQDVAKRRRLDPPTDVDAFSGSNRTNSRPQLHPTPSTPSVGNAPPTAPGLRAGLHPQLHPNLQAASSLGSGSSSREELPSGPRYQTQCVLGRGSFGTVCKAIEVRSRKTVAIKTVASSGAGREMEVLRRLSGNPNVVSLLGAFEGTDPEARTLNFVLEYIEDTLGRIIKHHRQQGTEMDFNFVRIYMYQLLRGLGSLYREGIVHRDIKPANLLVDPQSYCLKVCDFGTAKWVNTNEVSQAYVCSRFYRAPELILSTRDHNTSVDMWAAGCVLGEMLLHQPLFAGKDGIDQLFKIMEILGTPSNQQLSQMNPFYDSAAVFTYVPPLKWSKVLRARWSGQAESLLTMMLQYDPKSRPHPMEAMATDFFAELRKSPPKVRIASEFFNFTDQEMSSCKPELLRKLMPEKRPL* |
| Dinoflagellate-specific CDKD | *Cladocopium goreaui* | Liu *et al.* 2017^S25^ | >SymbC1.scaffold1699.6 SymbC1.scaffold1699:70847-80883(+) | MVNCDLSRTQDMDRAESLETAICAVQQAIGHIGLSSEWTSVLSSKLRRPCELCPDVRRVLEKNGWVRGQFRSPPVRELLLRELREMANAANSAHAASAANADHGQSLLPIRLDDDRRFERQYTFEEKEAPVGEGTYGAVYRAFCNLSKKTVAIKRVKMEHEDEGMPSTAIREVAVLKAADHPNVVKLLDVACSPGRLHLIFEFVDSNLKQYMKKFGLRLEAGVVRALHKQLMQGIDYCHARRIIHRDLKPQNILVDGQDNLKIADFGMARAFNLPIPKYTHEVVTTWYRPPEILFGCEDYSLGVDVWSAGCILGEMATGAALFHGDSEIDTIFQIFKKLGTPCEVEWPGLSELPDFKPSFPQWRKRPWSEIRNIVAQLGSAGTRLLDAMLRYDPLHRISARQTLLHEYFSVLDDVDANMTS |
| Unidentified CDK#1 | *Cladocopium goreaui* | MI population – Levin *et al.* 2016 ^S32^ | >C1MI_TR62820_c0_g1_i2_m.149847 | LAPRPPAARLDNPVSHQELTNNNCCKKRRSSFLLHHTMSRYEKIEKVGEGTFGVVYKAKDRQTGELVALKRMRLEAEEEGIPCTAIREISLLKELRHDNVVRLHDVVHSDRKLTLVFEFLQMDLRDYMDKAGEGGLDPWSVQHFMRQLLLGIEYCHYRMVLHRDLKPQNLLISRDRVLKLADFGLGRAFEIPVHRMTHDVVTLWYRPPDVLLGSTKYSCNIDIWSAGCIFAEMAIGHALFNGRNDSDQLLKIFTFLGTPTQTEWPSMMDCPHSSAMLARDALHESFKTKSVEDLLAMGGGFETLGALGCDLLLKMLQYEPQRRLCASEALAHPYFQQRL* |

**Table S5.** Selected unicellular marine organisms databases used for screening through for CDKs and cyclins.

| Taxa | **Species** | **Source of Database** | **Free-living/Symbiotic/Parasitic** | **Heterotroph or photosynthetic** |
| --- | --- | --- | --- | --- |
| Dinoflagellate | *Alexandrium fundyense* | MMETSP | Free-living | Photosynthetic |
|  | *Alexandrium monilatum* |  | Free-living | Photosynthetic |
|  | *Lingulodinium polyedra* |  | Free-living | Photosynthetic |
|  | *Karenia brevis* |  | Free-living | Photosynthetic |
|  | *Amphidinium cartecea* |  | Free-living | Photosynthetic |
|  | *Amphidinium* |  | Free-living | Photosynthetic |
|  | *Peridinium aciculiferum* |  | Free-living | Photosynthetic |
|  | *Pelagodinium bei* |  | Symbiotic | Photosynthetic |
|  | *Kryptoperdinium foliaceum* |  | Free-living | Photosynthetic |
|  | *Amoebophyra sp.* |  | Parasitic | Heterotroph |
|  | *Brandtodinium nutricula* |  | Symbiotic | Photosynthetic |
|  | *Polarella glacialis* |  | Free-living | Photosynthetic |
|  | *Ceratium fusus* |  | Free-living | Photosynthetic |
|  | *Ansanella granifera* | Ensembl ^S36^ | Free-living | Photosynthetic |
| Alveolate | *Perkinsus marinus* | NCBI ID:12737 | Parasitic | Heterotroph |
| Diatom | *Licmophora paradoxa* | MMETSP | Symbiotic | Photosynthetic |
|  | *Nitzschia puncata* |  | Free-living | Photosynthetic |
| Chlorophyta | *Chlorella variabilis* | NCBI ID:694 ^S37^ | Symbiotic | Photosynthetic |
|  | *Chlorella sorokiniana* | NCBI ID: 31394 ^S38^ | Free-living | Photosynthetic |
|  | *Chlorella vulgaris* | NCBI ID: 700^S39^ | Symbiotic | Photosynthetic |

**Table S6.** RNA-seq data taken from Maor‐Landaw *et al.*^S40^ of *Breviolum minutum* CDKs and cyclins that are differentially expressed (log_2_ fold-change) in free-living *versus* symbiotic states that correspond to CDK and cyclin proteins found in our study. Values < 0 indicate a down-regulation in expression, whereas values > 0 indicate an up-regulation in expression.

| **Symbiodiniaceae gene** | **log_2_ fold-change** | **Adjusted *p*-value** |
| --- | --- | --- |
| P/U-type Cyclin 1 | 0.34 | <0.001 |
| P/U-type Cyclin 2 | -0.54 | <0.001 |
| CDKB1 | 0.367 | 0.0019 |
| CDKB2 | 0.355 | < 0.0001 |
| CDKC | -0.6457 | < 0.0001 |
| CDKF | 0.9921 | < 0.0001 |
| CDKG | 0.398 | 0.01289 |
| CDKH | 0.697 | < 0.0001 |
| CDKI | -0.2309 | 0.0026 |
| CDK7-related protein | 0.363 | 0.0135 |
| CDK9 | 0.1817 | 0.03014 |

**References**

**S**1. Guindon, S. & Gascuel, O. A simple, fast, and accurate algorithm to estimate large phylogenies by maximum likelihood. *Syst. Biol.* **52**, 696–704 (2003).

S2. Letunic, I. & Bork, P. Interactive Tree Of Life (iTOL) v4: recent updates and new developments. *Nucleic Acids Res.* **47**, 256–259 (2019).

S3. Gavet, O. & Pines, J. Activation of cyclin B1-CDK1 synchronizes events in the nucleus and the cytoplasm at mitosis. *J. Cell Biol.* **189**, 247–259 (2010).

S4. Atikukke, G., Albosta, P., Zhang, H. & Finley Jr, R. L. A role for *Drosophila* Cyclin J in oogenesis revealed by genetic interactions with the piRNA pathway. *Mech. Dev.* **133**, 64–76 (2014).

S5. Yam, C. H., Fung, T. K. & Poon, R. Y. C. Cyclin A in cell-cycle control and cancer. *Cell. Mol. Life Sci. C.* **59**, 1317–1326 (2002).

S6. Malumbres, M. & Barbacid, M. Cell cycle, CDKs and cancer: a changing paradigm. *Nat. Rev. Cancer* **9**, 153–166 (2009).

S7. Maciejowski, J. *et al.* Mps1 directs the assembly of Cdc20 inhibitory complexes during interphase and mitosis to control M phase timing and spindle checkpoint signaling. *J. Cell Biol.* **190**, 89–100 (2010).

S8. Siu, K. T., Rosner, M. R. & Minella, A. C. An integrated view of cyclin E function and regulation. *Cell cycle* **11**, 57–64 (2012).

S9. Hinds, P. W. *et al.* Regulation of retinoblastoma protein functions by ectopic expression of human cyclins. *Cell* **70**, 993–1006 (1992).

S10. Wood, D. J. & Endicott, J. A. Structural insights into the functional diversity of the CDK-cyclin family. *Open Biol.* **8**, 180112 (2018).

S11. Sherr, C. J. & Roberts, J. M. CDK inhibitors: positive and negative regulators of G_1_ phase progression. *Genes Dev.* **13**, 1501–1512 (1999).

S12. Kim, J. K. & Diehl, J. A. Nuclear cyclin D1: an oncogenic driver in human cancer. *J. Cell. Physiol.* **220**, 292–296 (2009).

S13. Quelle, D. E. *et al.* Overexpression of mouse D-type cyclins accelerates G_1_ phase in rodent fibroblasts. *Genes Dev.* **7**, 1559–1571 (1993).

S14. Oakenfull, E. A., Riou-Khamlichi, C. & Murray, A. H. Plant D-type cyclins and the control of G_1_ progression. *Philos. Trans. R. Soc. London B Biol. Sci.* **357**, 749–760 (2002).

S15. Shupp, A., Casimiro, M. C. & Pestell, R. G. Biological functions of CDK5 and potential CDK5 targeted clinical treatments. *Oncotarget* **8**, 17373 (2017).

S16. Griffin, S. V, Olivier, J. P., Pippin, J. W., Roberts, J. M. & Shankland, S. J. Cyclin I protects podocytes from apoptosis. *J. Biol. Chem.* **281**, 28048–28057 (2006).

S17. Brinkkoetter, P. T. *et al.* Cyclin I activates CDK5 and regulates expression of Bcl-2 and Bcl-XL in postmitotic mouse cells. *J. Clin. Invest.* **119**, 3089–3101 (2009).

S18. Guevara, T., Sancho, M., Pérez-Payá, E. & Orzáez, M. Role of CDK5/cyclin complexes in ischemia-induced death and survival of renal tubular cells. *Cell cycle* **13**, 1617–1626 (2014).

S19. Morales, F. & Giordano, A. Overview of CDK9 as a target in cancer research. *Cell Cycle* **15**, 519–527 (2016).

S20. Guen, V. J. *et al.* CDK10/cyclin M is a protein kinase that controls ETS2 degradation and is deficient in STAR syndrome. *Proc. Natl. Acad. Sci.* **110**, 19525–19530 (2013).

S21. Malumbres, M. Cyclin-dependent kinases. *Genome Biol.* **15**, 122 (2014).

S22. Clijsters, L. *et al.* Cyclin F controls cell-cycle transcriptional outputs by directing the degradation of the three activator E2Fs. *Mol. Cell* **74**, 1264–1277 (2019).

S23. Malumbres, M. & Barbacid, M. Mammalian cyclin-dependent kinases. *Trends Biochem. Sci.* **30**, 630–641 (2005).

S24. Keeling, P. J. *et al.* The Marine Microbial Eukaryote Transcriptome Sequencing Project (MMETSP): illuminating the functional diversity of eukaryotic life in the oceans through transcriptome sequencing. *PLoS Biol.* **12**, e1001889 (2014).

S25. Liu, H. *et al.* *Symbiodinium* genomes reveal adaptive evolution of functions related to coral–dinoflagellate symbiosis. *Commun. Biol.* **1**, 1–11 (2018).

S26. Parkinson, J. E. *et al.* Gene expression variation resolves species and individual strains among coral-associated dinoflagellates within the genus *Symbiodinium*. *Genome Biol. Evol.* **8**, 665–680 (2016).

S27. Shoguchi, E. *et al.* Draft assembly of the *Symbiodinium minutum* nuclear genome reveals dinoflagellate gene structure. *Curr. Biol.* **23**, 1399–1408 (2013).

S28. Aranda, M. *et al.* Genomes of coral dinoflagellate symbionts highlight evolutionary adaptations conducive to a symbiotic lifestyle. *Sci. Rep.* **6**, 39734 (2016).

S29. Bayer, T. *et al.* *Symbiodinium* transcriptomes: genome insights into the dinoflagellate symbionts of reef-building corals. *PLoS One* **7**, e35269 (2012).

S30. Ladner, J. T., Barshis, D. J. & Palumbi, S. R. Protein evolution in two co-occurring types of *Symbiodinium*: an exploration into the genetic basis of thermal tolerance in *Symbiodinium* clade D. *BMC Evol. Biol.* **12**, 217 (2012).

S31. González-Pech, R. A., Vargas, S., Francis, W. & Wörheide, G. Transcriptomic Resilience of a Coral Holobiont to Low pH. *Front. Mar. Sci.* **12**, 403 (2017).

S32. Levin, R. A. *et al.* Sex, scavengers, and chaperones: transcriptome secrets of divergent *Symbiodinium* thermal tolerances. *Mol. Biol. Evol.* **33**, 2201–2215 (2016).

S33. Xiang, T., Nelson, W., Rodriguez, J., Tolleter, D. & Grossman, A. R. *Symbiodinium* transcriptome and global responses of cells to immediate changes in light intensity when grown under autotrophic or mixotrophic conditions. *Plant J.* **82**, 67–80 (2015).

S34. Lin, S. *et al.* The *Symbiodinium kawagutii* genome illuminates dinoflagellate gene expression and coral symbiosis. *Science (80-. ).* **350**, 691–694 (2015).

S35. Shoguchi, E. *et al.* Two divergent *Symbiodinium* genomes reveal conservation of a gene cluster for sunscreen biosynthesis and recently lost genes. *BMC Genomics* **19**, 458 (2018).

S36. Jang, S. H., Jeong, H. J., Chon, J. K. & Lee, S. Y. *De novo* assembly and characterization of the transcriptome of the newly described dinoflagellate *Ansanella granifera*: Spotlight on flagellum-associated genes. *Mar. Genomics* **33**, 47–55 (2017).

S37. Blanc, G. *et al.* The *Chlorella variabilis* NC64A genome reveals adaptation to photosymbiosis, coevolution with viruses, and cryptic sex. *Plant Cell* **22**, 2943–2955 (2010).

S38. Arriola, M. B. *et al.* Genome sequences of *Chlorella sorokiniana* UTEX 1602 and *Micractinium conductrix* SAG 241.80: implications to maltose excretion by a green alga. *Plant J.* **93**, 566–586 (2018).

S39. Wakasugi, T. *et al.* Complete nucleotide sequence of the chloroplast genome from the green alga *Chlorella vulgaris*: the existence of genes possibly involved in chloroplast division. *Proc. Natl. Acad. Sci.* **94**, 5967–5972 (1997).

S40. Maor‐Landaw, K., van Oppen, M. J. H. & McFadden, G. I. Symbiotic lifestyle triggers drastic changes in the gene expression of the algal endosymbiont *Breviolum minutum* (Symbiodiniaceae). *Ecol. Evol.* **10**, 451–466 (2020).
