# Supplementary figures and images for: Phylogenetic analysis of cell-cycle regulatory proteins within the Symbiodiniaceae
Source: Sci Rep. 2020 Nov 24;10:20473. doi: 10.1038/s41598-020-76621-1 (PMC7686383; doi:10.1038/s41598-020-76621-1)

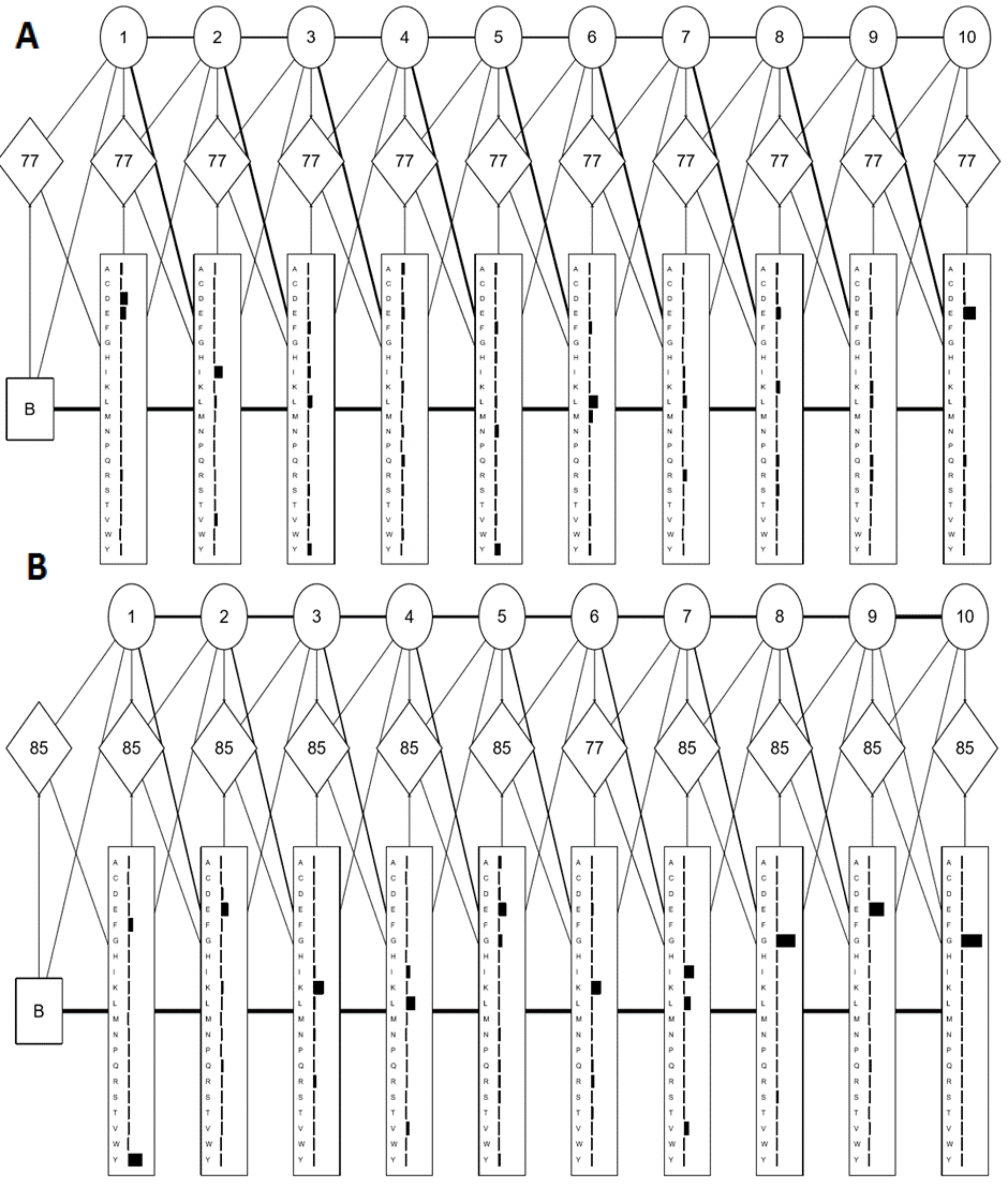

Supplement: Supplementary file 5 — Supplementary Information 2. [file 41598_2020_76621_MOESM5_ESM.png]

Tree scale: 0.1

SH-value  
01

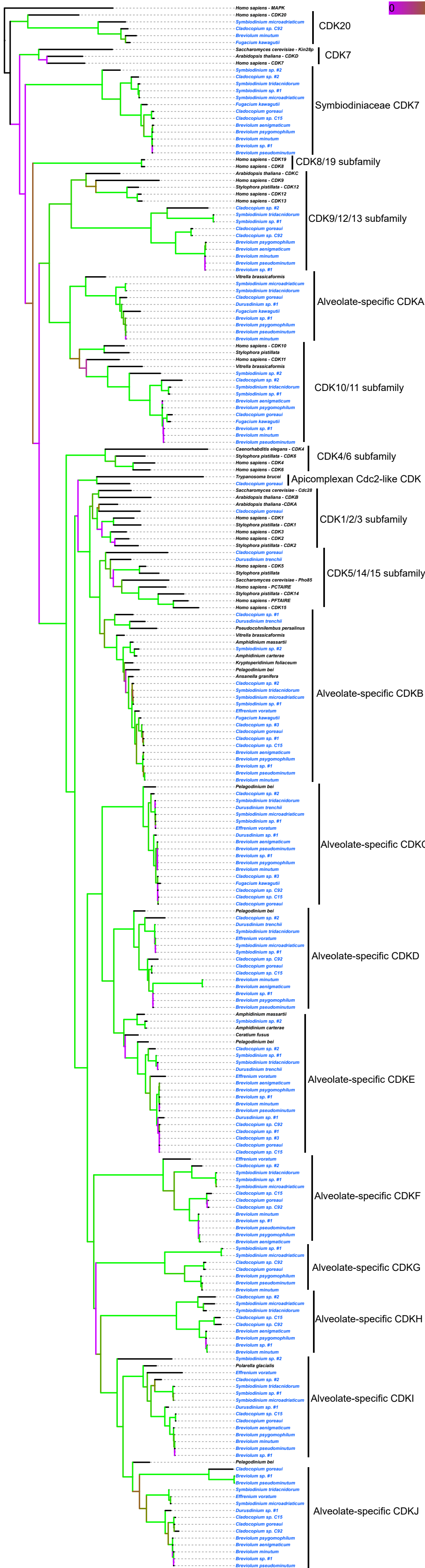

Supplement: Supplementary file 6 — Supplementary Figure 2. [file 41598_2020_76621_MOESM6_ESM.pdf]

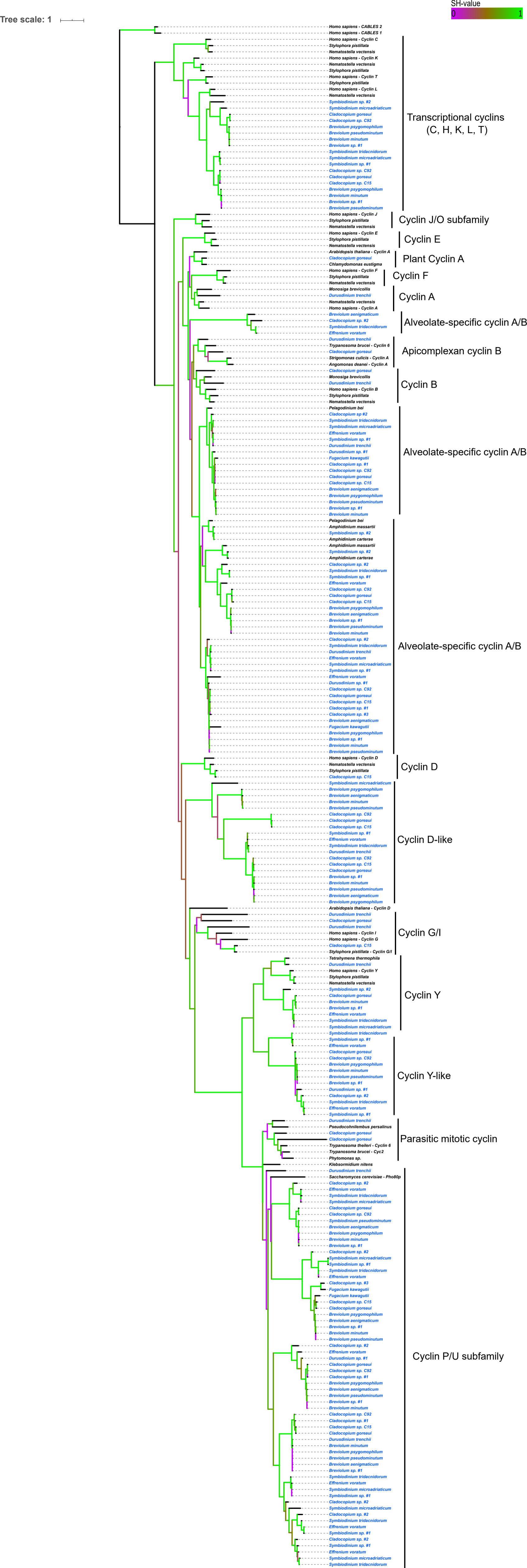

Supplement: Supplementary file 7 — Supplementary Figure 3. [file 41598_2020_76621_MOESM7_ESM.pdf]
